# Supplementary material for: A conserved chronobiological complex times C. elegans development
Source: EMBO J. 2025 Oct 20;44(22):6368–96. doi: 10.1038/s44318-025-00585-z (PMC12624140; doi:10.1038/s44318-025-00585-z)
Supplement: Supplementary file 1 — Appendix [file 44318_2025_585_MOESM1_ESM.pdf]

Table of contents

Page 2: Appendix Figure S1. PER proteins have a crucial role in mammalian circadian rhythms.

Page 3: Appendix Figure S2. LIN-42 and KIN-20 isoforms

Page 4: Appendix Figure S3. LIN-42 N-terminus does not bind to biotinylated CK1 *in vitro.*

Page 5: Appendix Figure S4. *kin-20(D310A)* affects molt timing.

Page 6: Appendix Table S1. *lin-42* mutant phenotypes.

Page 7: Appendix Table S2. Strain and genotype details/

Page 10. Appendix Table S3. Oligo details.

Page 14. Appendix Table S4. crRNA details.

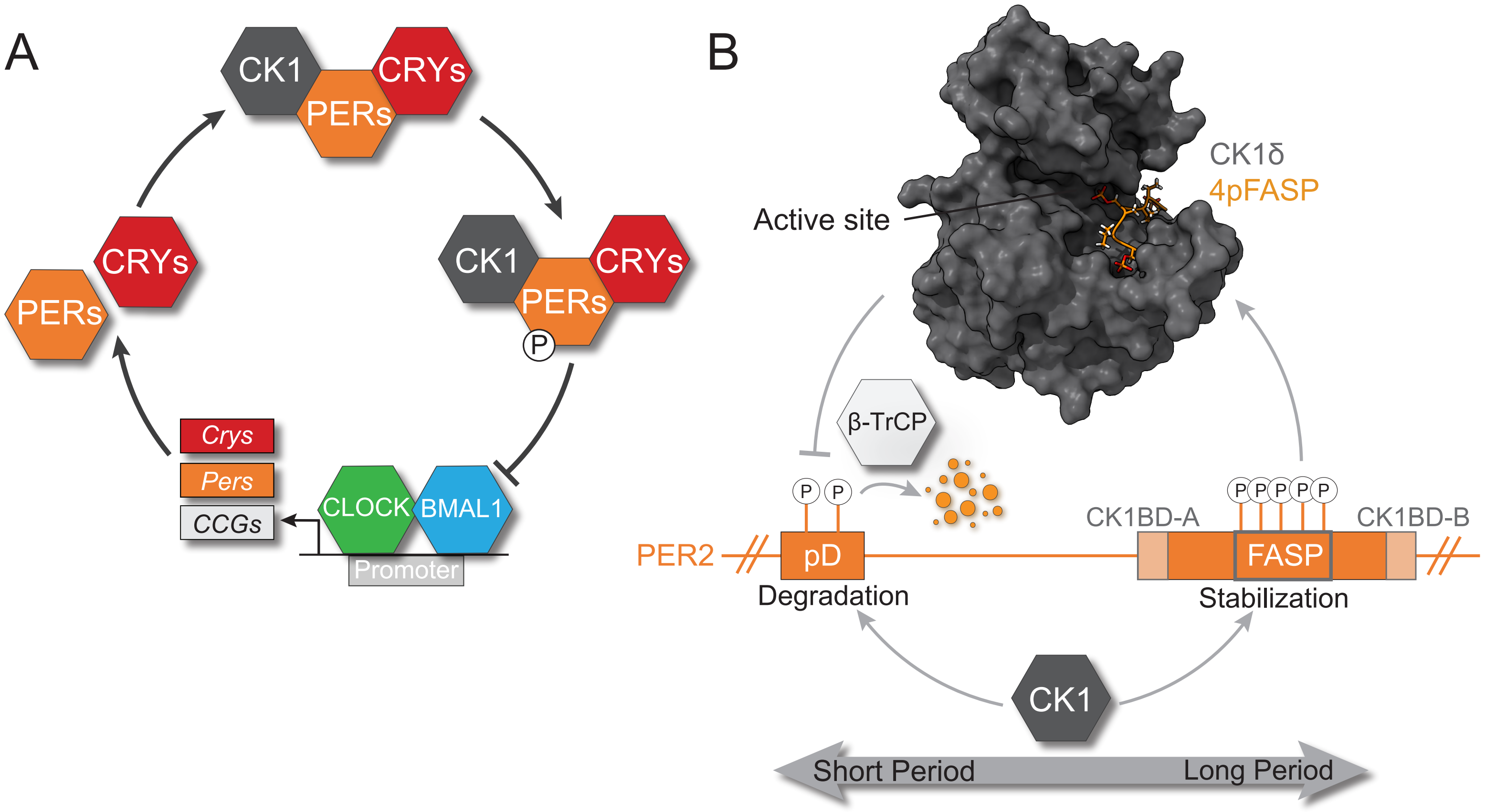

Appendix Figure S1 PER proteins have a crucial role in mammalian circadian rhythms.
(A) Cartoon schematic of the primary transcription-translation feedback loop that generates ~24-hour rhythms in mammals. CCGs, Clock-controlled genes. (B) Cartoon schematic illustrating the mammalian phosphoswitch that dictates CK1-dependent regulation of PER stability. Crystal structure of human CK1δ (gray) bound to phosphorylated PER2 FASP (orange, 4pFASP) peptide, PDB: 8d7o. pD, phosphodegron; FASP, Familial Advanced Sleep Phase.

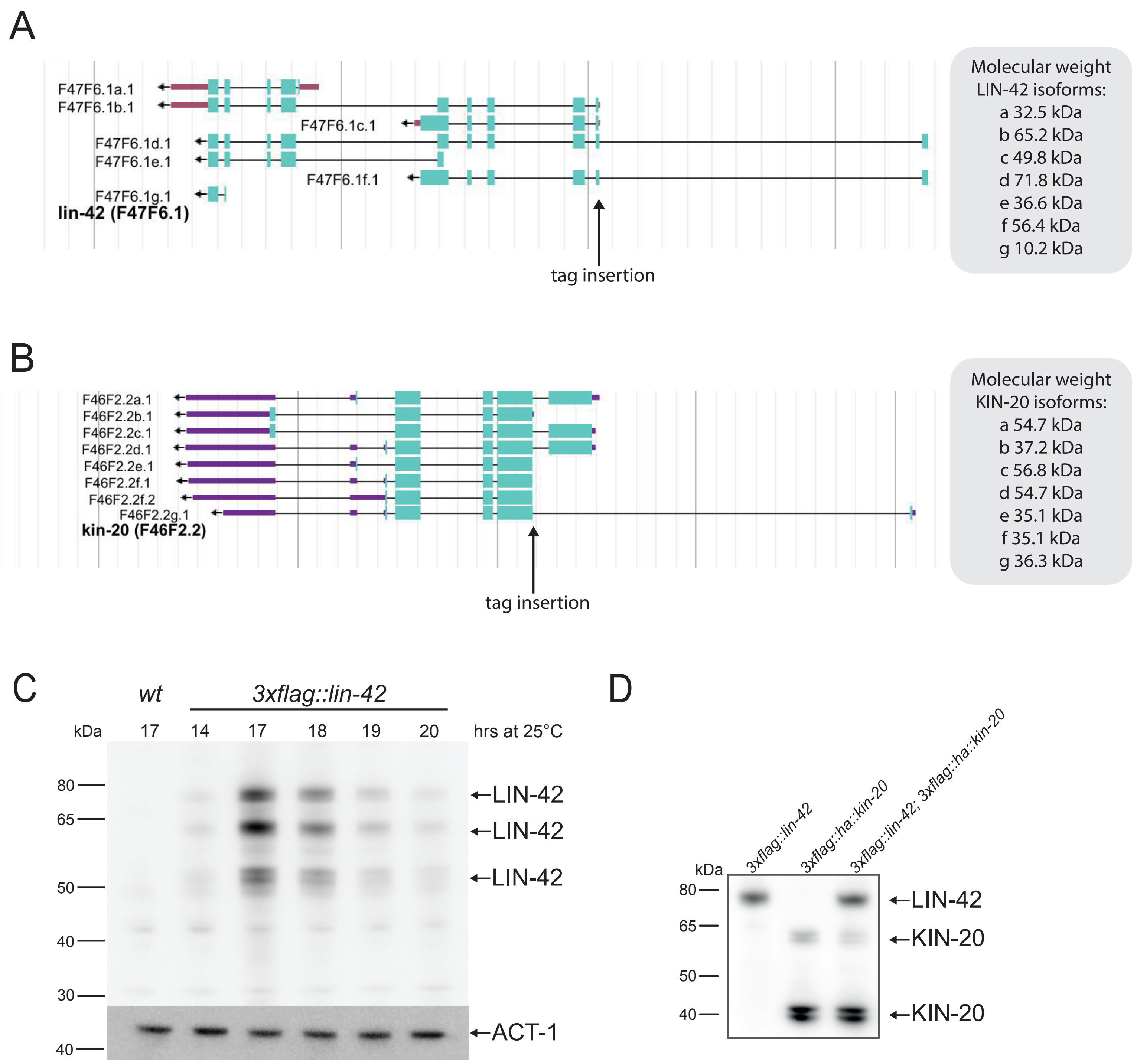

Appendix Figure S2 LIN-42 and KIN-20 isoforms.
(A) *lin-42* locus with isoforms a-g indicated. Image adapted from JBrowse2 genome browser (wormbase.org). The insertion of the tag is indicated with an arrow. **(B)** *kin-20* locus with isoforms a-g indicated. Image adapted from JBrowse2 genome browser (wormbase.org). Calculated isoform size in kilodaltons (kDa) is provided in A and B. The insertion of the tag is indicated with an arrow. **(C)** Western Blot with extracts of wild type (wt) and *lin-42(xe321[3xflag::lin-42])* animals. Synchronized animals collected at indicated times after plating (grown at 25°C). Top part of the blot probed with anti-FLAG-HRP (1:1,000), lower part probed with anti-actin (1:7,500). Bands for LIN-42 and ACT-1 are indicated with an arrow. **(D)** Western Blot with extracts of mixed stage animals expressing *lin-42(xe321[3xflag::lin-42])*, *kin-20(xe328[3xflag::ha::kin-20])* or both. Blot probed with anti-FLAG-HRP (1:1,000). Arrows indicate bands for LIN-42 and KIN-20. Two biological repeats were performed for C and D.

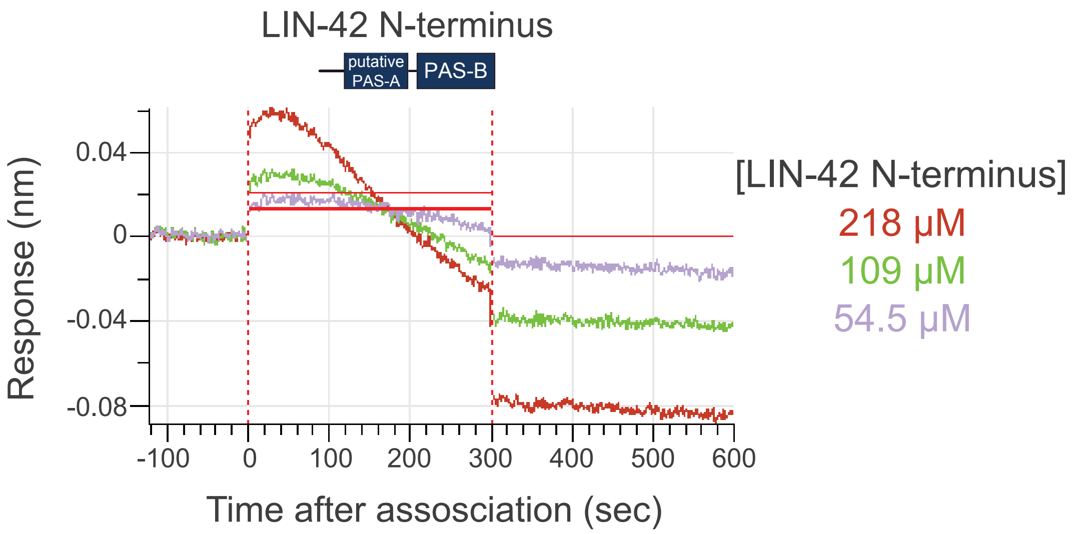

Appendix Figure S3 LIN-42 N-terminus does not bind to biotinylated CK1 *in vitro.*
Bio-layer interferometry (BLI) data for indicated LIN-42 protein binding to immobilized, biotinylated CK1. Inset values represent the concentrations of LIN-42 for individual binding reactions. Data shown from one representative experiment of n ≥ 3 assays.

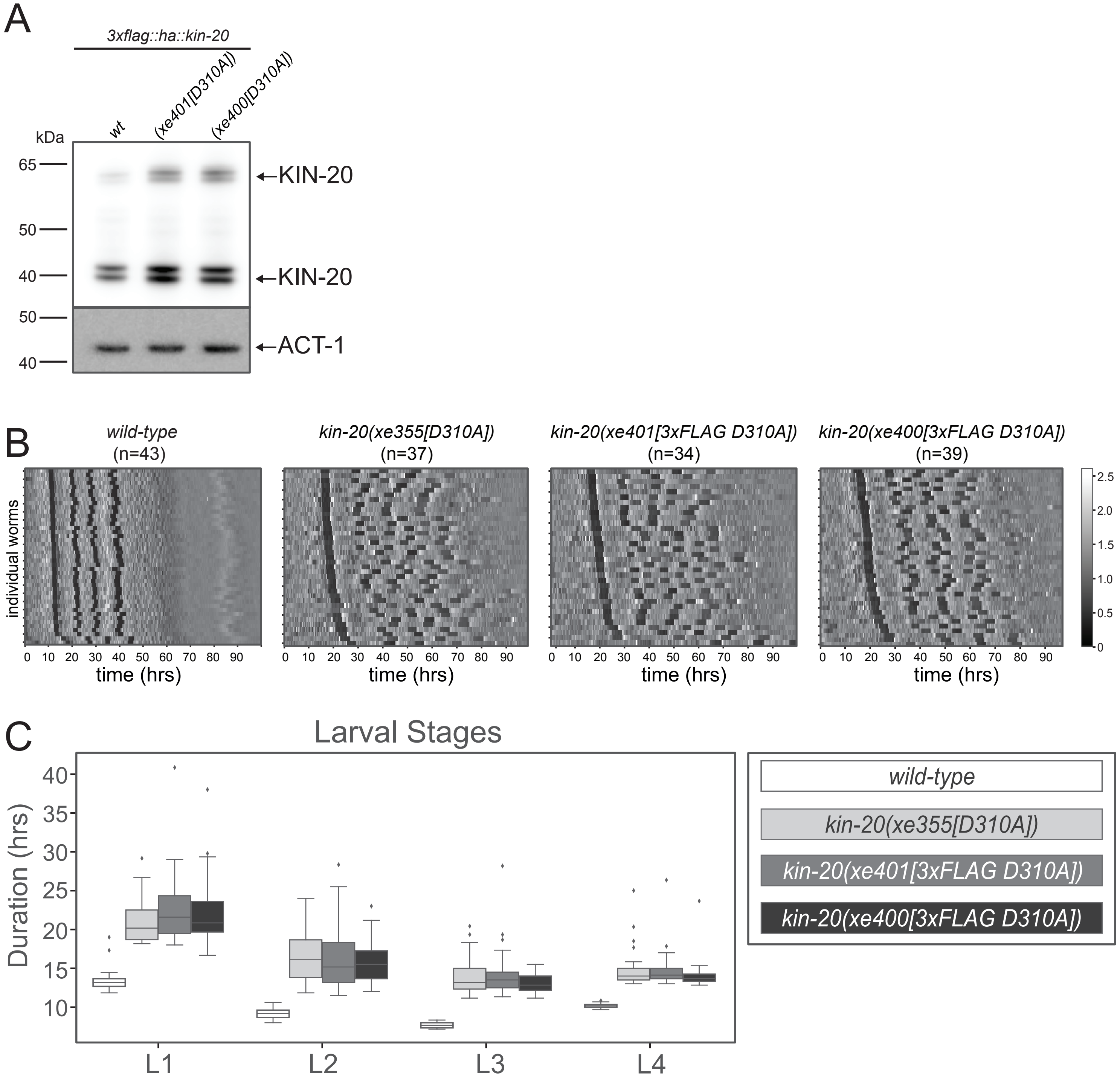

Appendix Figure S4 *kin-20(D310A)* affects molt timing.
(A) Western Blot with extracts from *3xflag::kin-20* wild type, *(xe401[D310A])* and *(xe400[D310A])* mutant animals. Top panel probed with anti-FLAG-HRP (1:1,000). Lower panel probed with anti-actin-1 (1:7,500). Arrows indicate bands for KIN-20. Two biological repeats were performed. **B)** Heatmaps showing trend-corrected luminescence traces from the indicated genotype. Each horizontal line represents one animal. Traces are sorted by entry into the first molt. Darker color indicates low luminescence signal and corresponds to the molts. Two biological repeats were performed. **(C)** Boxplots showing the duration (in hours) for the larval stage from the luciferase assay. Statistics were done using the Mann-Whitney U-test. Stars indicate the significance of difference between the Wt strain and the different lin-42 mutant animals: * p<0.05, ** p<0.01, *** p<0.001, **** p<0.0001. Boxplots were generated using the boxplot function in python's seaborn package (v0.13.2) using the default options (center = median, boxes represent values within the 0.25 (Q1) and 0.75 (Q3) quantiles (the interquantile distance or IQR), whiskers represent values within Q1 - 1.5IQR and Q3 + 1.5IQR, and extrema are the minima and maxima for each condition).

Appendix Table S1 ***lin-42*** **mutant phenotypes**

Strain	Allele	Short name	Arrest[a] (%)	Precocious alae[b] (%)			Animals that reached adulthood	
				Total	Complete	In-complete	BOW[c] (%)	Brood size[d]
N2	*wild-type*		0	0	0	0	0	313 ± 25
MT2257	*lin-42(n1089)*		0	94.4	72.2	22.2	34.4	180 ± 58
RB1843	*lin-42(ok2385)*		48.1	80	40	40	85	nd*
VC398	*kin-20(ok505)*		42.5	0	0	0	52.2	52 ± 21
JDW335	*lin-42(wrd63)*	ΔCK1BD	28.9	6.8	0	6.8	1	149 ± 41
JDW577	*lin-42(wrd179)*	ΔCK1BD	27.3	7.7	0	7.7	5	159 ± 31
JDW439	*lin-42(wrd107)*	ΔTail	0	56.3	5.4	50.9	29.1	261 ± 58
JDW579	*lin-42(wrd181)*	ΔTail	0	43.4	0	43.4	18.5	270 ± 39
JDW340	*lin-42(wrd67)*	ΔPAS	0	13.5	0	13.5	1.5	251 ± 38
JDW648	*lin-42(wrd227)*	ΔPAS	0	24.6	1.8	22.8	0	283 ± 30

Animals grew from eggs hatched onto seeded plates at low density after sodium hypochlorite treatment. $n \geq 20$ for all analyses. [a]Percentage of animals that failed to reach adulthood by day 8 after hatching or died as young larvae [b]Percentage of animals with alae formation in early L4 (L4.0-L4.2). [c]Percentage of animals that exhibited bag-of-worms phenotype. [d]Average number of progeny from fertile adults; arrested and bagged animals were not included in the calculation.*Broods were not counted in animals with high instance of BOW phenotype

Appendix Table S2 Strains and genotypes

Strain Name	*Genotype*	Short name	Source	Experiment used	Figure	Outcrossed	growth condition
JDW335	*lin-42(wrd63[ΔCK1BD]) II*	*lin-42ΔCK1BD*	This work	Phenotypic analysis	1C-F	X3	OP50
JDW439	*lin-42(wrd107[ΔTail]) II*	*lin-42ΔTail*	This work	Phenotypic analysis	5B-E	X2	OP50
JDW340	*lin-42(wrd67[ΔPAS]) II*	*lin-42ΔPAS*	This work	Phenotypic analysis	1C-F	X2	OP50
RB1843	*lin-42(ok2385)*	*lin-42(ok2385)*	CGC	Phenotypic analysis	1C-F	n/a	NGM 2% plates, OP50
MT2257	*lin-42(n1089)*	*lin-42(n1089)*	CGC	Phenotypic analysis	1C-F	n/a	NGM 2% plates, OP50
VC398	*kin-20(ok505)*	*kin-20(0)*	CGC	Phenotypic analysis	5B-E	n/a	NGM 2% plates, OP50
HW1993	*xeSi312 [eft-3p::luc::gfp::unc-54 3' UTR, unc-119(+) in oxTi177] IV*	*luciferase* reporter strain	This work	Luciferase assay (control)	1B, 5A, EV1, EV3 Appendix S4	X3	liquid culture in OP50/S-Basal
JDW409	*lin-42(wrd67) II; xeSi312 [eft-3p::luc::gfp::unc-54 3' UTR, unc-119(+) in oxTi177] IV*	*lin-42ΔPAS in luciferase background*	This work	Luciferase assay	1B, EV1A-D	n/a	liquid culture in OP50/S-Basal
JDW417	*lin-42(wrd63) II; xeSi312 [eft-3p::luc::gfp::unc-54 3' UTR, unc-119(+) in oxTi177] IV*	*lin-42(ΔCK1BD) in luciferase background*	This work	Luciferase assay	1B, EV1A-D	n/a	liquid culture in OP50/S-Basal
JDW590	*lin-42(wrd107) II; xeSi312 [eft-3p::luc::gfp::unc-54 3' UTR, unc-119(+) in oxTi177] IV*	*lin-42(ΔTail) in luciferase background*	This work	Luciferase assay	5A, EV3A-E	n/a	liquid culture in OP50/S-Basal
JDW658	*lin-42(ok2385) II xeSi312 [eft-3p::luc::gfp::unc-54 3' UTR, unc-119(+) in oxTi177] IV*	*lin-42(ok2385) in luciferase background*	lin-42(ok2385) from CGC (RB1843) crossed to LucIV	Luciferase assay	1B, EV1A-D	n/a	liquid culture in OP50/S-Basal
HW3730	*lin-42(n1089) II; xeSi312 [eft-3p::luc::gfp::unc-54 3' UTR, unc-119(+) in oxTi177] IV*	*lin-42(n1089) in luciferase background*	lin-42(n1089) from CGC (MT2257) crossed to LucIV	Luciferase assay	1B, EV1A-D	n/a	liquid culture in OP50/S-Basal
HW3368	*xeSi312 [eft-3p::luc::gfp::unc-54 3' UTR, unc-119(+) in oxTi177] IV; kin-20 (ok505) X*	*kin-20(0) in luciferase background*	kin-20(ok505) from CGC (VC398) crossed to LucIV	Luciferase assay	5A, EV3A-D	n/a	liquid culture in OP50/S-Basal

HW3484	*xeSi312 [eft-3p::luc::gfp::unc-54 3' UTR, unc-119(+) in oxTi177] IV, kin-20(xe355[D310A]) X*	*kin-20 D310A in luciferase background*	This work	Luciferase assay	5A, EV3, Appendix S4B,C	n/a	liquid culture in OP50/S-Basal
HW3479	*lin-42 (xe321[3xflag::lin-42])II, kin-20(xe328[3xflag::ha::kin-20]) X*	*3xflag::lin-42, 3xflag-ha::kin-20*	this work	WB, HA-IP	2C,D, Appendix S2D	n/a	NGM 2% plates, OP50
HW3303	*lin-42(xe315[gfp::tev::3xflag::lin-42]) II; kin-20(xe329[wrmScarlet::tev::linker::kin-20]) X*	*gfp::lin-42; wrmScarlet::kin-20*	This work	Imaging	6B	n/a	NGM 2% plates, OP50
HW1008	*EG6701, xeSi55[dpy-30p::sart-3::gfp::his::flag::xrn-2 3', unc-119(+)] I*	*sart-3::gfp::flag*	Rügger et. al	IP-MS (control)	2A	n/a	NGM 2% plates, OP50
HW3270	*lin-42(xe321[3xflag::lin-42]) II*	*3xflag::lin-42*	This work	IP-MS, Phospho mapping IP	2A, 4F, Appendix S2C,D	X3	NGM 2% plates, OP50
HW3859	*xeSi312 [eft-3p::luc::gfp::unc-54 3' UTR, unc-119(+)in oxTi177] IV; kin-20(xe401[3xflag::ha::kin-20 D310A]) X*	*kin-20(xe401[3xFLAG D310A]) in luciferase background*	This work	WB, Luciferase Assay	Appendix S4B,C	X3	NGM 2% plates, OP50
HW3858	*xeSi312 [eft-3p::luc::gfp::unc-54 3' UTR, unc-119(+)in oxTi177] IV; kin-20(xe400[3xflag-ha::kin-20 D310A]) X*	*kin-20(xe400[3xFLAG D310A]) in luciferase background*	This work	WB, Luciferase Assay	Appendix S4B,C	X3	NGM 2% plates, OP50
HW3451	*bchSi84[eft-3p::gfp1-10(codon-optimized)::tbb-2 3UTR]) II; kin-20[(xe354[3xflag::4xgfp11]) X*	*splitgfp::kin-20*	This work	Imaging	6A, 6C-E	X3	NGM 2% plates, OP50
JDW792	*lin-42 (wrd63)II; bchSi84 (pIK407[Peft-3::gfp1-10(codon-optimized)::tbb-2 3UTR]) II; Kin-20[(xe354[3xflag::4xgfp11::kin-20]) X*	*lin-42(ΔCK1BD) in splitgfp::kin-20 background*	This work	Imaging	6C+E	n/a	NGM 2% plates, OP50
HW3293	*kin-20(xe328[3xflag::ha::kin-20])*	*3xflag-ha::kin-20*	This work	WB	Appendix S2D, S4A	X3	NGM 2% plates, OP50
IFM217	*bchSi84 (pIK407[Peft-3::gfp1-10(codon-optimized)::tbb-2 3UTR]) II*		This work	cross		X3	NGM 2% plates, OP50

HW3892	*lin-42(wrd107) II; bchSi84 (pIK407[Peft-3::gfp1-10(CO)])II; kin-20(xe354[3xflag::4xgfp11]) X*	*lin-42(ΔTail), kin-20::splitgfp*	This work	Imaging	6D-E, EV4	n/a	NGM 2% plates, OP50

Appendix Table S3. Oligos used
Repair templates for CRISPR-Cas 9 injections

Oligo name	sequence (5'-3')	Purpose
6920	ACTGACCCGAGAAGCACTGACACTGCACACTAAACGGTTCGAGGATGAATATAAGGACACTTGGTGCAGACTCCGAGATTCTCAGAATTAATAAGCTACTGCCCA	CRISPR repair template for *lin-42(ΔTail)*
6158	GAAAGTTGCCAGCGCCCCGCCGACCACCTCACTTGGTGCAGGTGAGAGAATTTTCTGAGTTATTT	CRISPR repair templatefor *lin-42(ΔCK1BD)*
5783	CACTTGCACCAGCAATGCGTGAGGAAGGTGCCACGCTCAAGGATCAGAACCAGGGCTTCCCGGCCAACAT	CRISPR repair template for *lin-42(ΔPAS-A/B)*
KK107	GAATAATATATATTCAAATTTTCAGCGGAGATGGACTATAAAGACGATGATGACAAAGATTACAAGGACGACGACGACAAAGACTACAAAGATGATGACGACAAGGGAGGTGGAGGTGGAGCTTACCCATACGATGTTCCAGATTACGCTGGAGGTGGAGGTGGAGCTGAACTTCGTGTCGGCAATCGTTTCCGCCTCGG	CRISPR repair template for *kin-20::3xFLAG-HA*

KK90	TCCCGCTATTTTCCTATTAAAATCTTTCTTCAACTCTTATTTTATTCCAGAACGTGGCACCATCAGCCAAATGGACTACAAAGACCATGACGGTGATTATAAAGATCATGACATCGATTACAAGGATGACGATGACAAGGAGCCAGCCGGGCACTCAAGCGCAACACATAACATCGTTGTGCCCAACGCCAATCCCACGC	CRISPR repair template for *3xFLAG::lin-42*
KK137	TGAAAACCGTCCTGCTGCTTGCCGATCAAATGTTGTCTCGTGTGGAATTTATTCATTGCCGAGATTACATTCATCGCGCGATTAAGCCGGATAACTTTTTAATGGGTCTTGGAAAACGAGGAAATCTGGTCTATGTAAGTTTTTCTTTTGTGAGGGATTAGCCAGCCTACTATGTTGTGCCTTTTTTTGCAGATTATTGA	CRISPR repair template for *kin-20 D310A*

Primers for amplification of repair template from a plasmid

Oligo number	Forward primer (5'-3')	Reverse primer (5'-3')	Purpose

KK41/KK42	TGCTTTTAAAACCAAATTTCCCGCTATTTTCCTATTAAAATCTTTCTTCAACTCTTATTTTATTCCAGAACGTGGCACCATCAGCCAAATGAGTAAAGGAGAAGAACTTTTCACTGGAGT	CGCGAGGAGCTAGGCAGGCTAGAAGGCAAACTGTACCTGCGTGGGATTGGCGTTGGGCACAACGATGTTATGTGTTGCGCTTGAGTGCCCGGCTGGCTCCATGCTTCCGCCGGTACCTCC	Oligos with overhangs to lin-42 to amplify GFP::TEV::3xFLAG from plasmid pIK384
KK102/KK103	CTTCTTCATTTGTTTGAATATTTTGACCCAAGTAGATGTCACCGAACGAGCCGCTTCCGATTTTGCGACCGAGGCGGAAACGATTGCCGACACGAAGTTCGCTTCCGCCGGTACCTCCAC	GCTTTACGTCAATGTCAGAGCGATTTGAAATCTAAAGTGCAAAATTCACAATACCAGATTTATGAATTGTGAATAATATATATTCAAATTTTCAGCGGAGATGGTCAGCAAGGGAGAGGC	Oligos with overhangs to kin-20 to amplify wrmScarlet::TEV::Linker from plasmid pIK385
KK145/KK146	GTGAATAATATATATTCAAATTTTCAGCGGAGATGAGTACCTCCGGCGGATCCGG	CGACCGAGGCGGAAACGATTGCCGACACGAAGTTCGCTTCCGCCGGTACCTCCAC	Oligos with overhangs to kin-20 to amplify 4xGFP11::Linker::FLAG from plasmid pIK401

Genotyping primers

Oligo number	Forward primer (5'-3')	Reverse primer (5'-3')	Reverse primer (5'-3')	Purpose
4529/4530	CATCTTGCCATCATCACCAC	TGGGTTCCGATAGAATTTGG		Genotyping oligos for *lin-42CKBD* deletions
5790/5791	CCAGTCCCTTTTGCCTGGAT	TGGGTTCCGATAGAATTTGGCAT		Genotyping oligos for *lin-42CKBD* deletions
6921/ 6922	GGGAGGCAGTGTGTCAAAAC	GGCTTGAATGTTTGGGCCTG		Genotyping oligos for *lin-42Tail* deletions
5784/5785/5786	GTCCTGAATTGGCCTGAAAA	ATTCTCTCACCTGCACCAAG	ACCGTGGGGTAATGTGAAGG	Genotyping oligos for *lin-42PAS* deletions
KK43/KK44	CTGTGTGAAGTTTTGGCATCT	CTTCCTCACGCATTGCTG		Genotyping oligos for xe321 and xe315

KK104/KK105	AGATACCTCCA GTTCCGC	TCGAGTGAAGG GCCTAGT		Genotyping oligos for xe328, xe354 and xe329
KK117/KK139	TTCGTGTCGGC AATCGTTT	ATTCGCTCATTT CGGCTC		Genotyping oligos for xe364. PCR product needs to be digested with NruI (only cuts in wt)

Plasmids

plasmid name	Backbone	Insert	comments
pIK384	cloning vector	linker::gfp::tev::3x flag	Used to amplify gfp::tev::3xflag with primers KK41/KK42 to generate xe315
pIK385	cloning vector	wrmScarlet::tev::li nker::aid::3xflag	Used to amplify wrmScarlet::tev: :linker with primers KK102/KK103 to generate xe329
pIK401	cloning vector	4xgfp11::linker::te v::3xflag	Used to amplify 4xgfp11::linker:: flag with primers KK145/KK146 to generate xe354
pIK407	pCFJ150	eft-3p::gfp1-10 (codon optimized)::tbb-2 3`UTR	Used to generate IFM217
pMM002	pMM001 (Meeuse et. al (2020)	eft- 3p::luc::gfp::unc- 54 3' UTR	Used to generate HW1993. Plasmid source Meeuse et. al (2020)

Appendix Table S4. crRNAs used

Number	Target	5'-3' crRNA sequence (Pam not included)
143	*lin-42ΔCK1BD*	GAGTGGTGGGTCCGTTGAGG
144	*lin-42ΔTail*	CCTCCTCTCTCCTAATGCTA
145	*lin-42ΔCK1BD*	AAGACGAGTACAAGGACACT
146	*lin-42ΔPAS*	GGTGTTCGGGGGTGACAACG
147	*lin-42ΔPAS*	CCACCATCACTCAAGCCTCA
359	*lin-42ΔTail*	GTACTCGTCTTCAAATCGCT
KK136	*kin-20* D310A	TTGATGGGTCTTGGAAAGCG
KK99	*kin-20* tagging	GCGGAGATGGAACTTCGTGT
KK50	*lin-42* tagging	TCAGCCAAATGGAGCCAGCC
