# Supplementary figures and images for: A conserved chronobiological complex times C. elegans development
Source: EMBO J. 2025 Oct 20;44(22):6368–96. doi: 10.1038/s44318-025-00585-z (PMC12624140; doi:10.1038/s44318-025-00585-z)

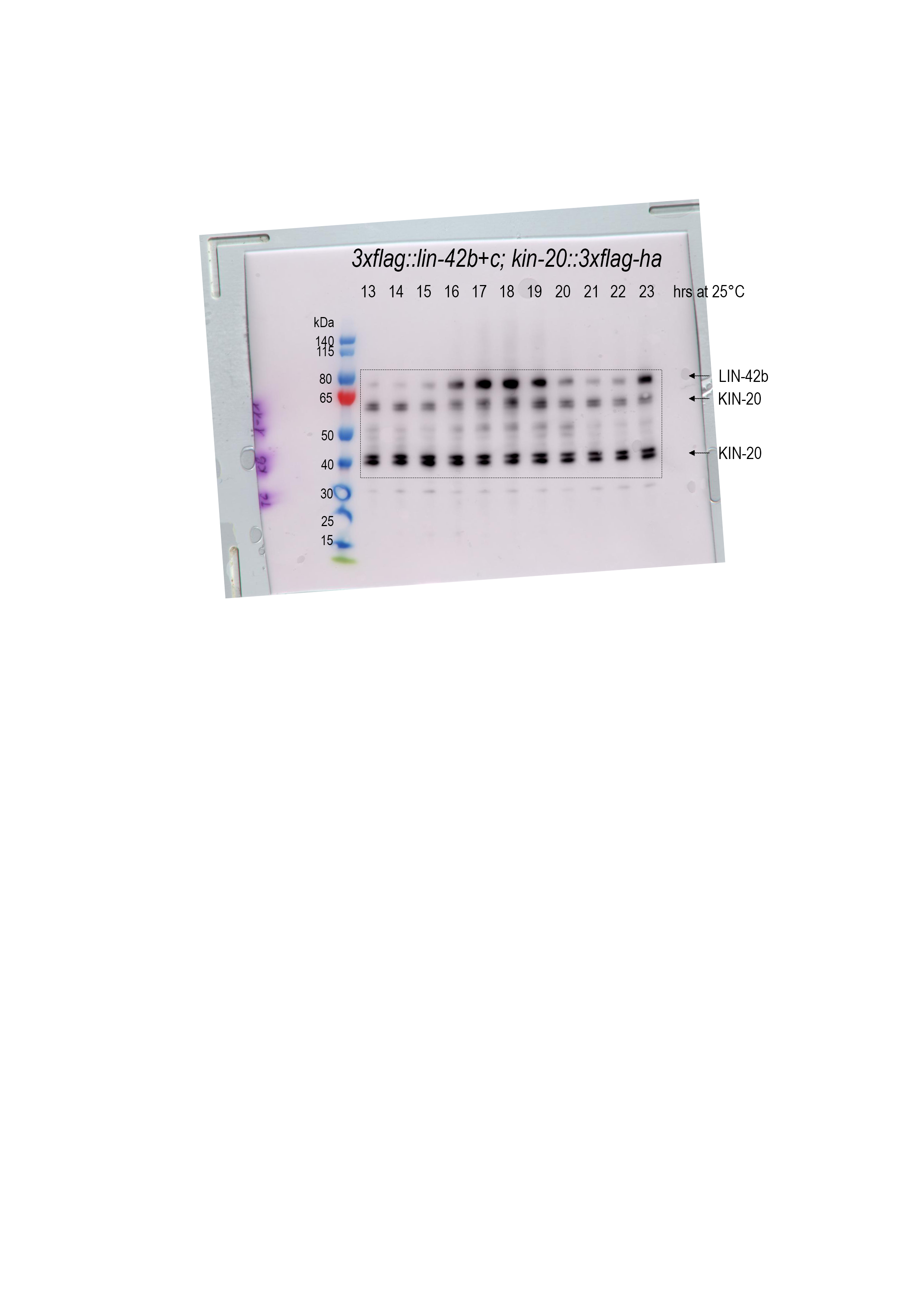

Supplement: Supplementary file 4 — Source data Fig. 2 [file 44318_2025_585_MOESM4_ESM.zip › Figure 2/2C/Western_FLAG_details.tif]

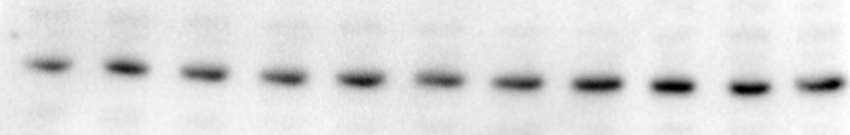

Supplement: Supplementary file 4 — Source data Fig. 2 [file 44318_2025_585_MOESM4_ESM.zip › Figure 2/2C/Western_Actin_Cropped.tif]

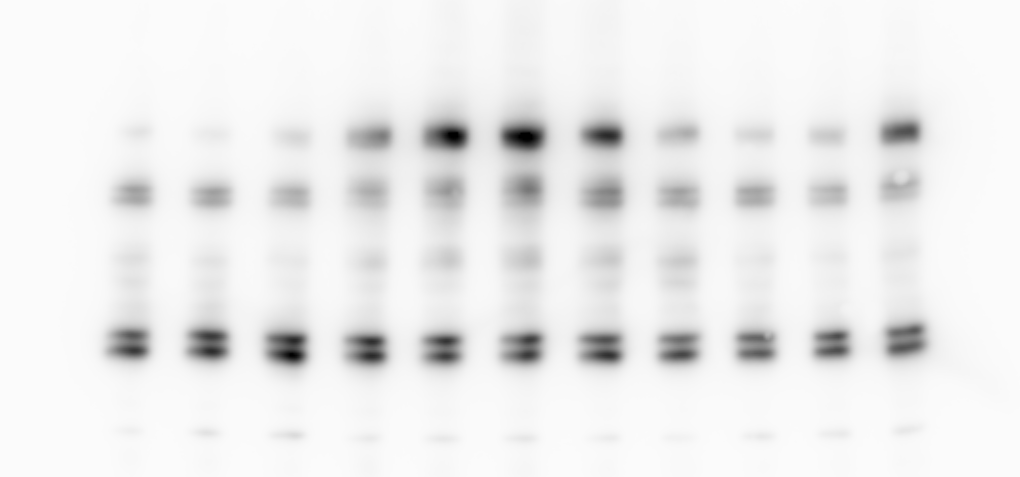

Supplement: Supplementary file 4 — Source data Fig. 2 [file 44318_2025_585_MOESM4_ESM.zip › Figure 2/2C/Western_FLAG_Cropped.tif]

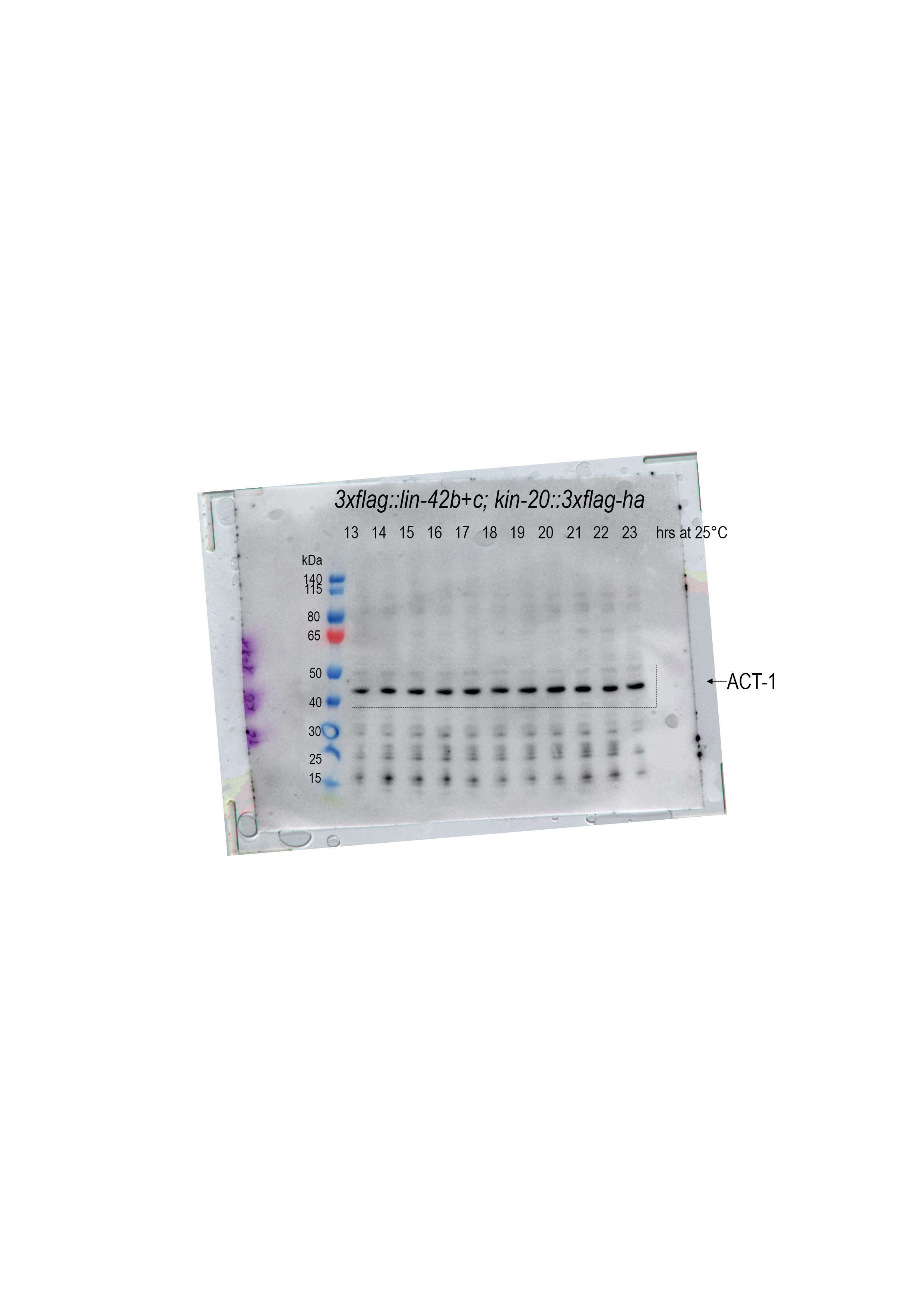

Supplement: Supplementary file 4 — Source data Fig. 2 [file 44318_2025_585_MOESM4_ESM.zip › Figure 2/2C/Western_Actin_Details.tif]

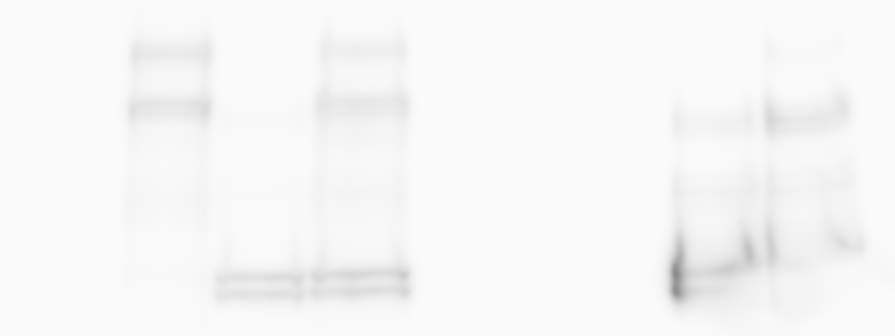

Supplement: Supplementary file 4 — Source data Fig. 2 [file 44318_2025_585_MOESM4_ESM.zip › Figure 2/2D/Western FLAG_cropped.tif]

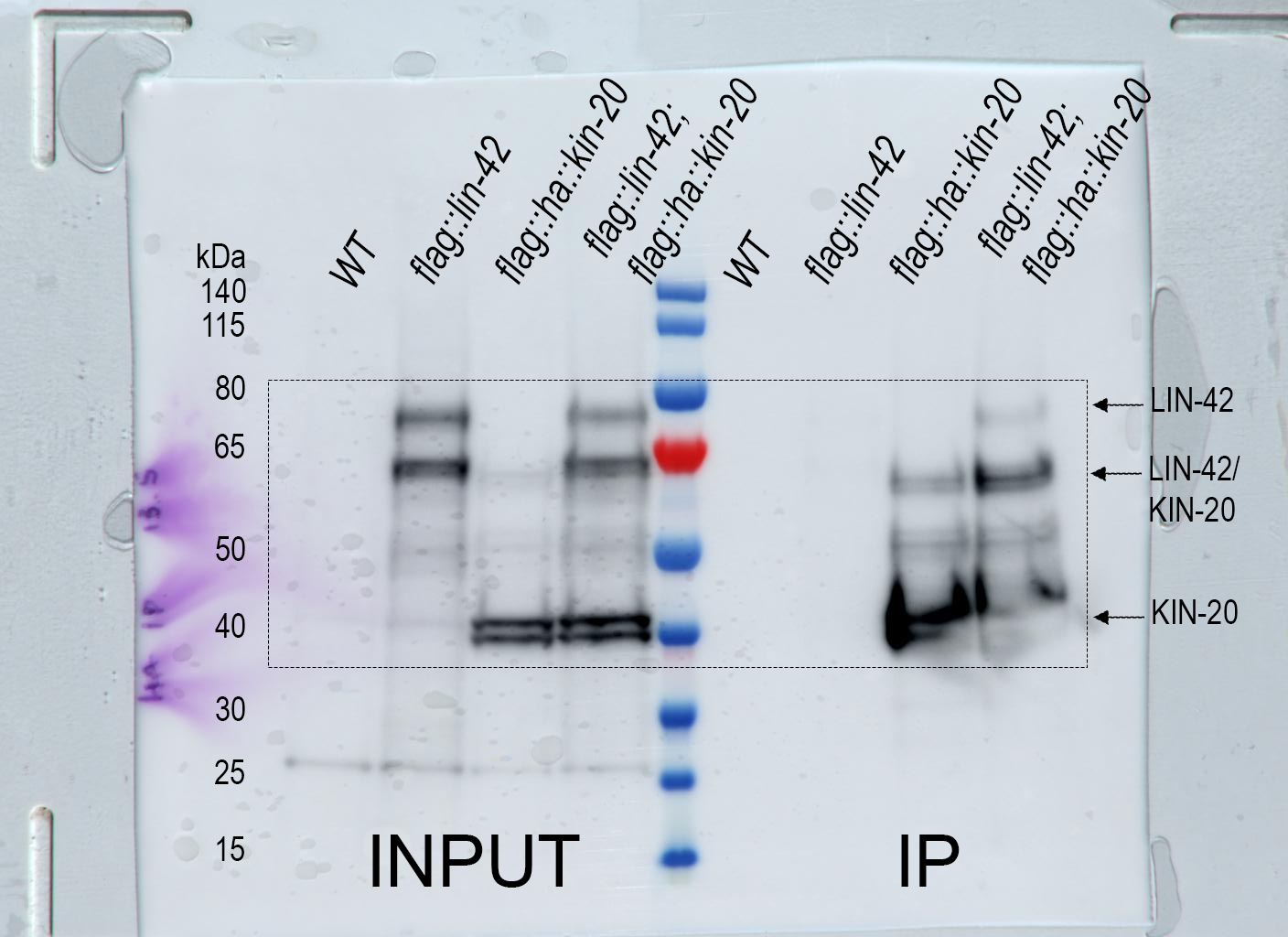

Supplement: Supplementary file 4 — Source data Fig. 2 [file 44318_2025_585_MOESM4_ESM.zip › Figure 2/2D/Western_FLAG_Details.tif]

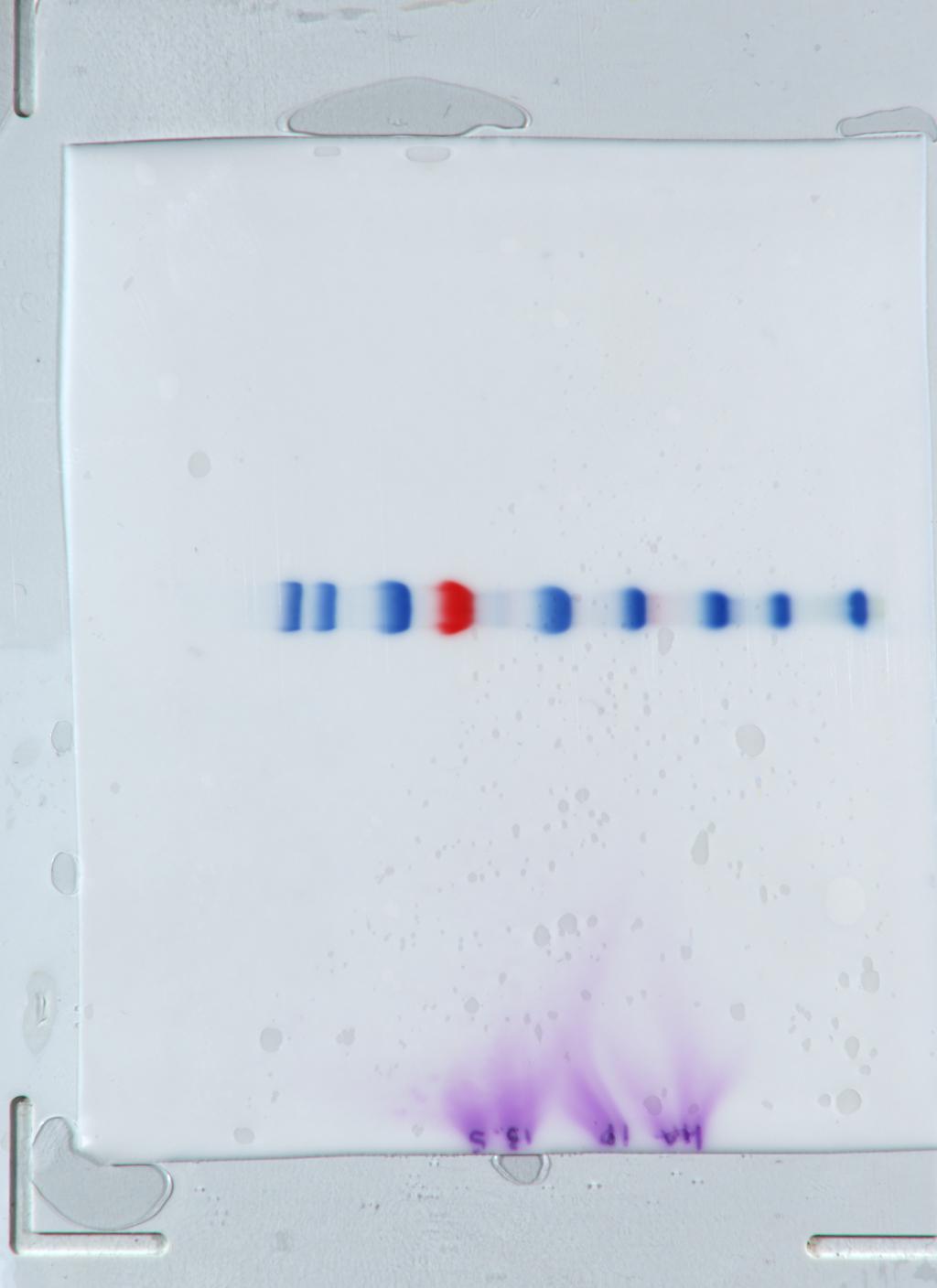

Supplement: Supplementary file 4 — Source data Fig. 2 [file 44318_2025_585_MOESM4_ESM.zip › Figure 2/2D/raw images/15052025_150s 2025.05.13_16.05.02_Ch-Marker.jpg]

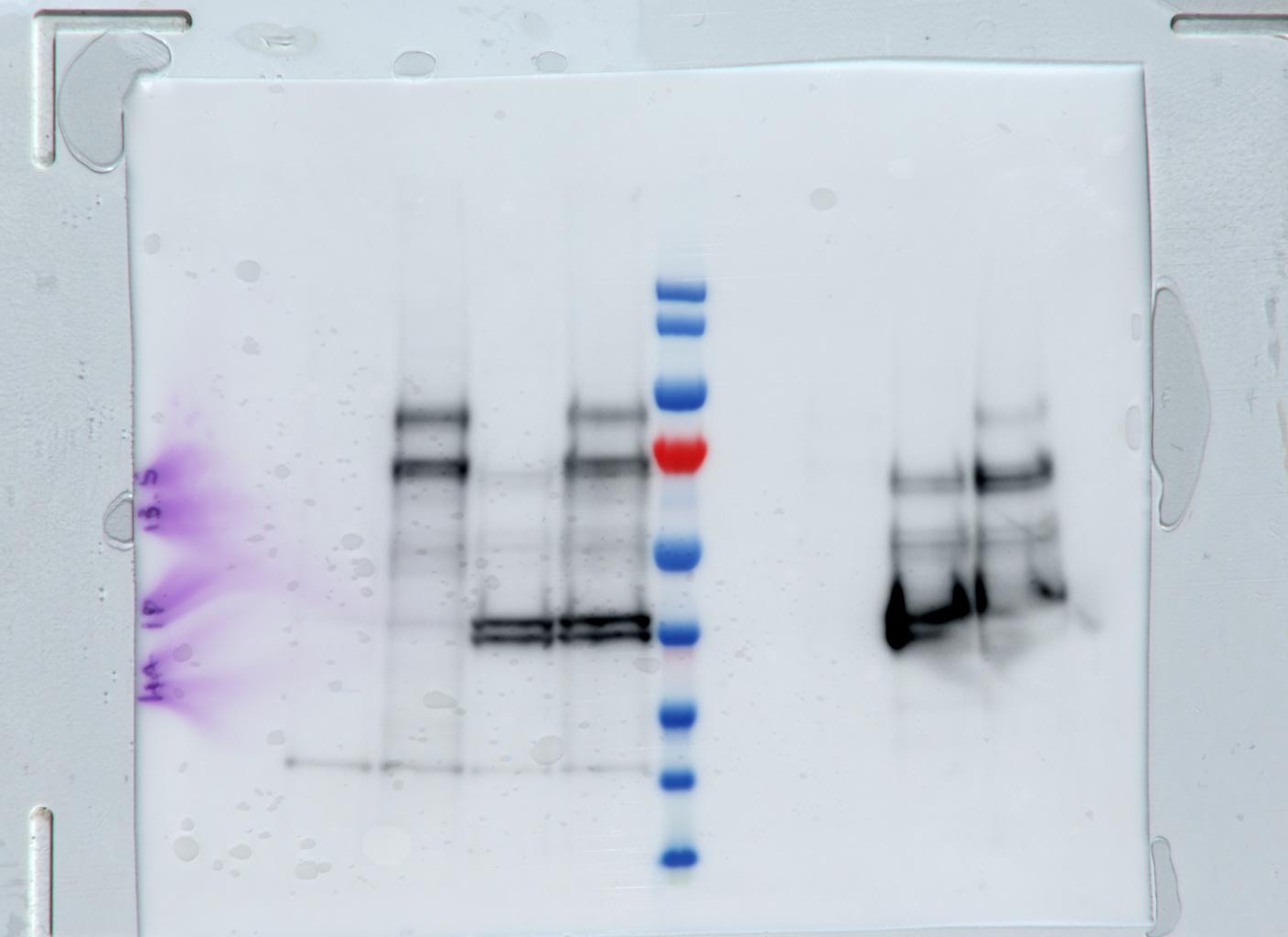

Supplement: Supplementary file 4 — Source data Fig. 2 [file 44318_2025_585_MOESM4_ESM.zip › Figure 2/2D/raw images/15052025_150s 2025.05.13_16.05.02_Ch+Marker.jpg]

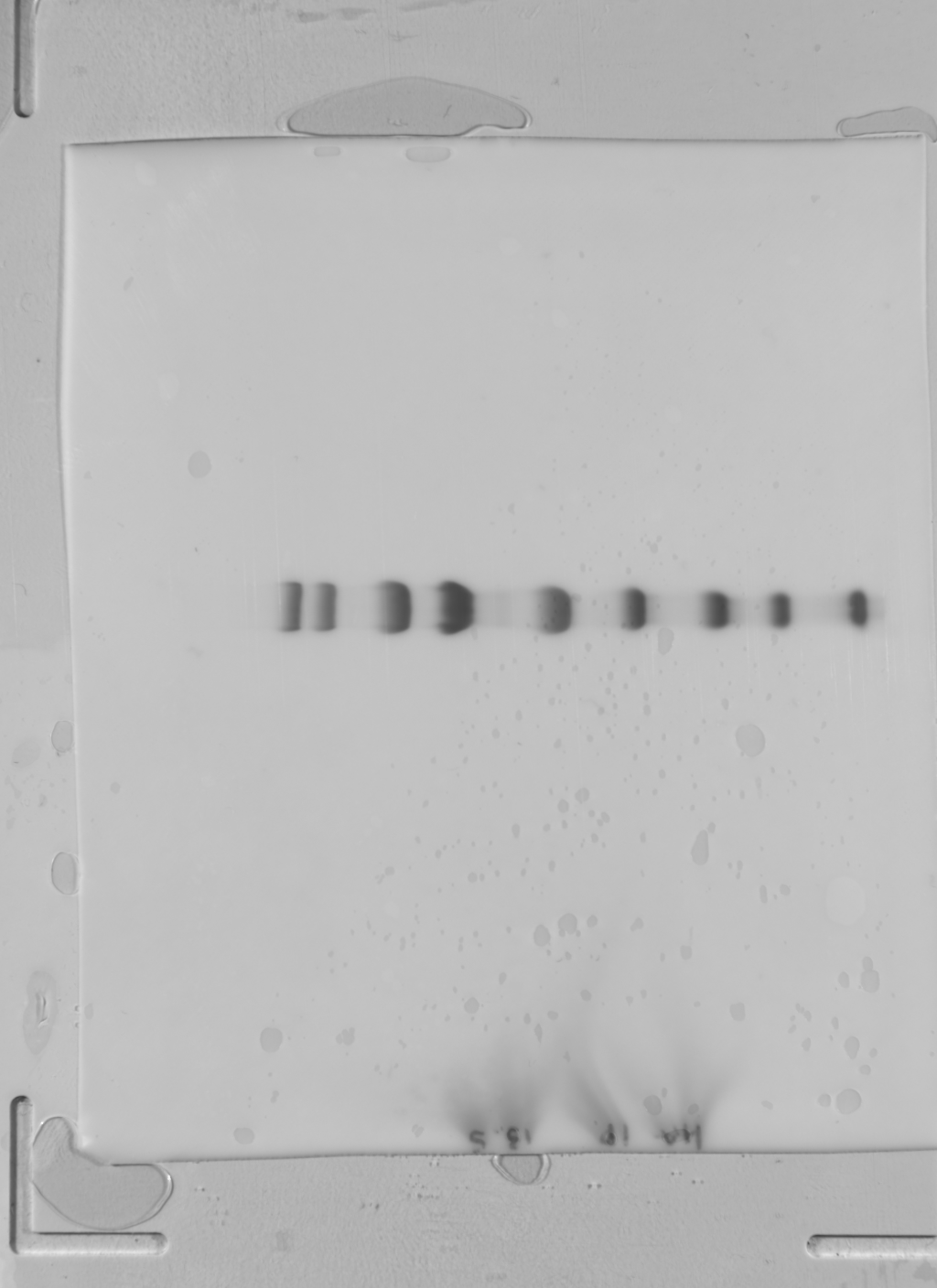

Supplement: Supplementary file 4 — Source data Fig. 2 [file 44318_2025_585_MOESM4_ESM.zip › Figure 2/2D/raw images/15052025_150s 2025.05.13_16.05.02_Ch-Marker.tif]

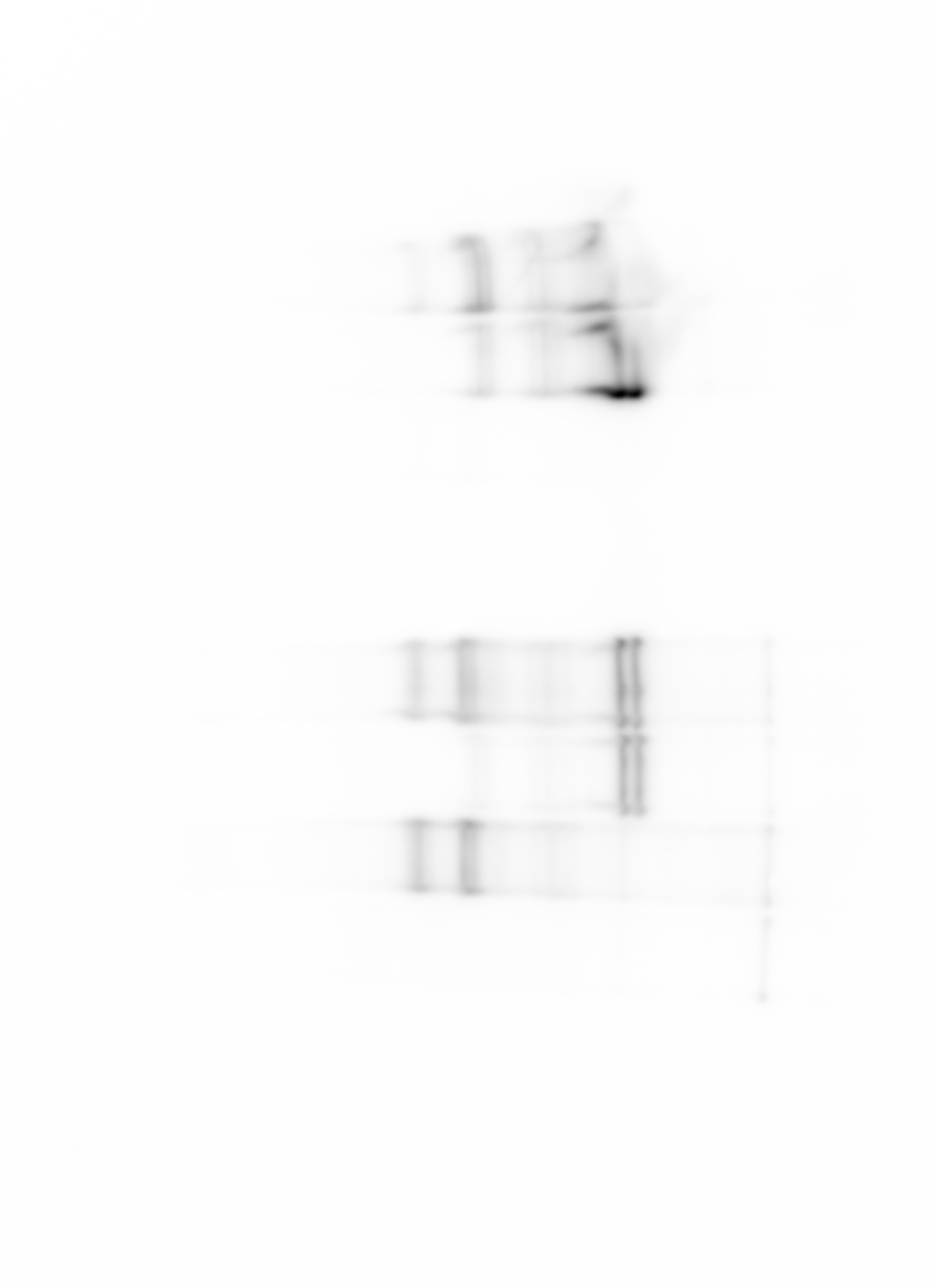

Supplement: Supplementary file 4 — Source data Fig. 2 [file 44318_2025_585_MOESM4_ESM.zip › Figure 2/2D/raw images/15052025_150s 2025.05.13_16.05.02_Ch.tif]

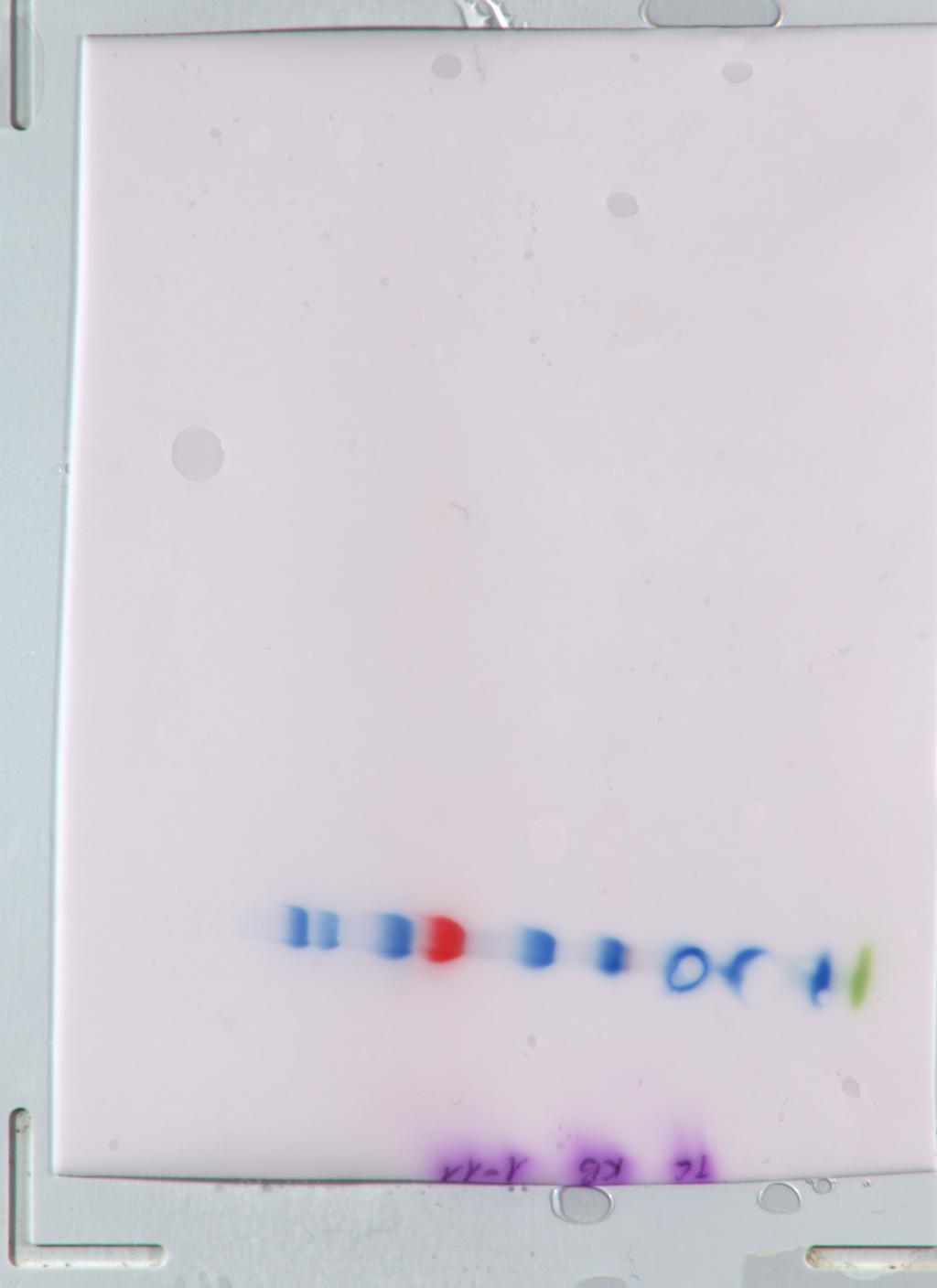

Supplement: Supplementary file 4 — Source data Fig. 2 [file 44318_2025_585_MOESM4_ESM.zip › Figure 2/2C/Western Images/FLAG/20230201_Lin42Kin20_10s 2023.02.01_05.41.18_Ch-Marker.jpg]

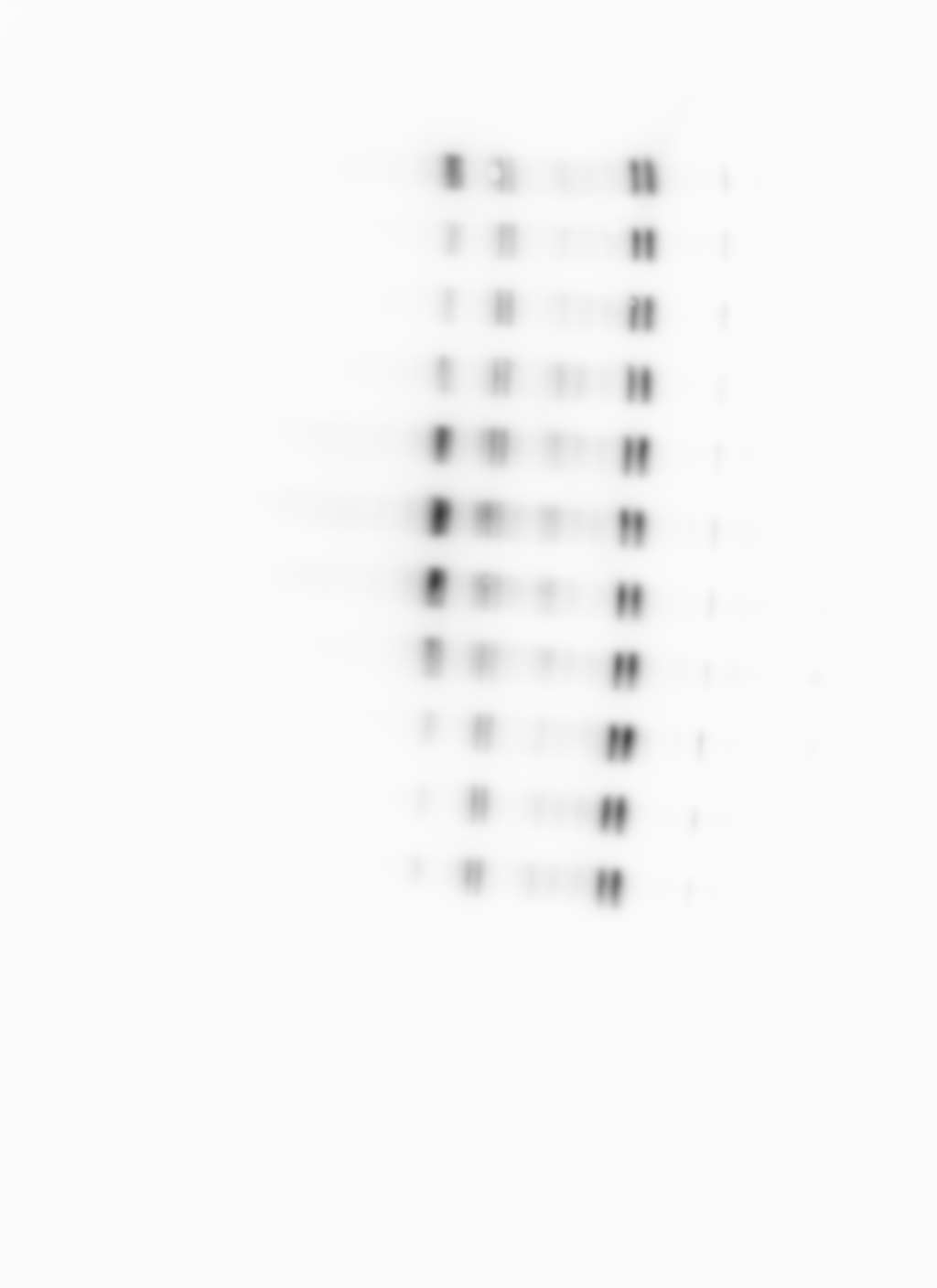

Supplement: Supplementary file 4 — Source data Fig. 2 [file 44318_2025_585_MOESM4_ESM.zip › Figure 2/2C/Western Images/FLAG/20230201_Lin42Kin20_10s 2023.02.01_05.41.18_Ch.tif]

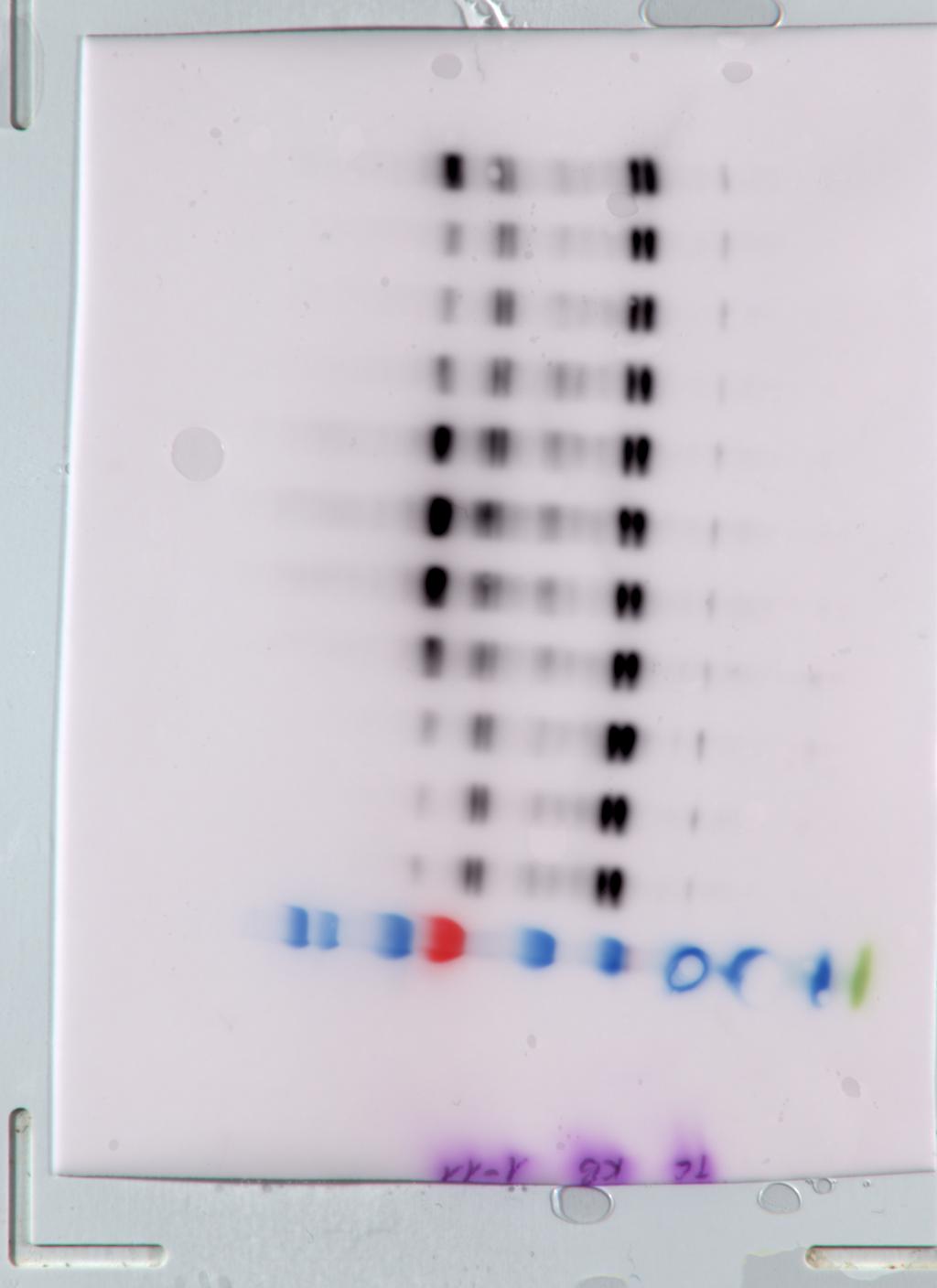

Supplement: Supplementary file 4 — Source data Fig. 2 [file 44318_2025_585_MOESM4_ESM.zip › Figure 2/2C/Western Images/FLAG/20230201_Lin42Kin20_10s 2023.02.01_05.41.18_Ch+Marker.jpg]

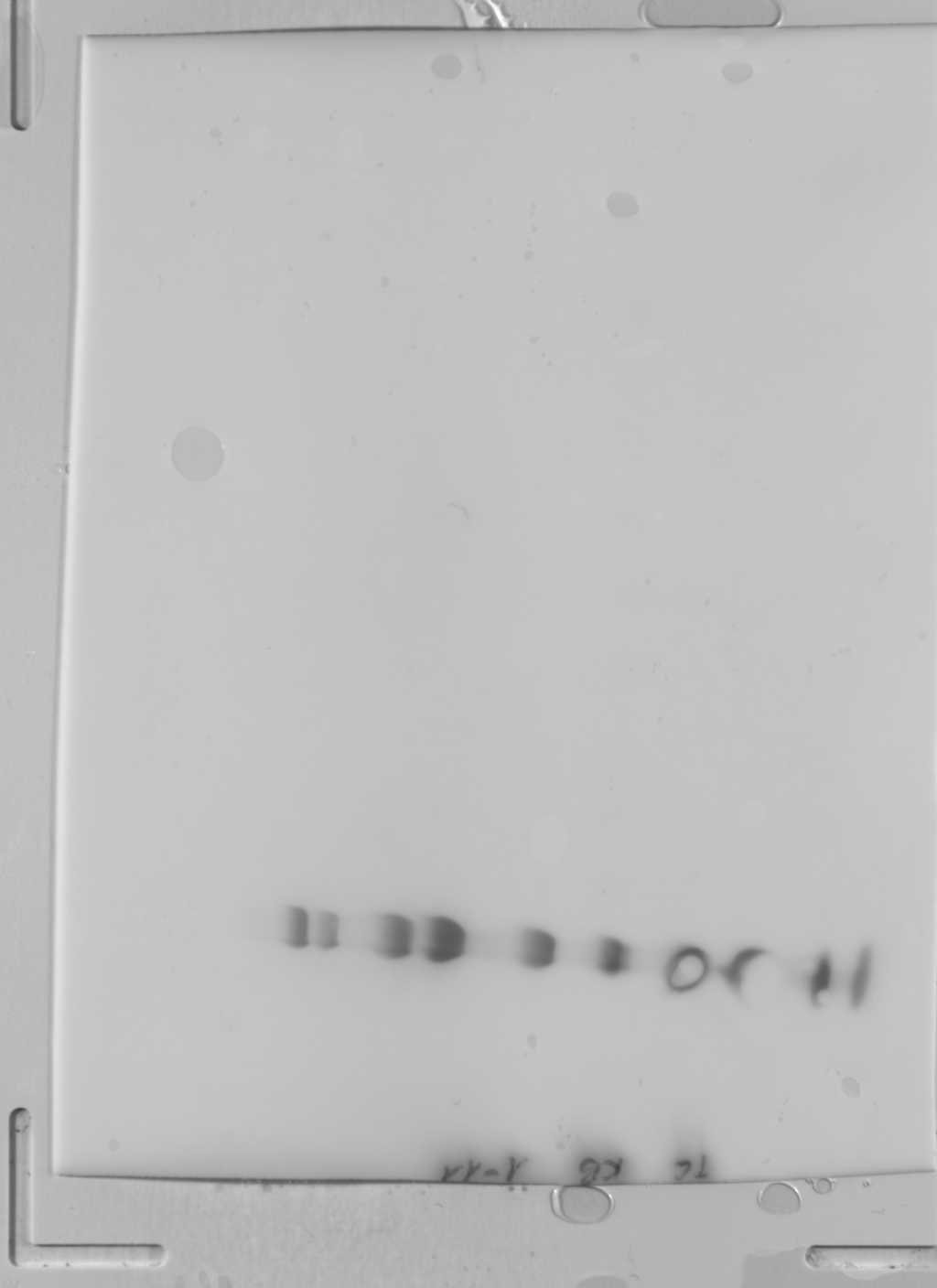

Supplement: Supplementary file 4 — Source data Fig. 2 [file 44318_2025_585_MOESM4_ESM.zip › Figure 2/2C/Western Images/FLAG/20230201_Lin42Kin20_10s 2023.02.01_05.41.18_Ch-Marker.tif]

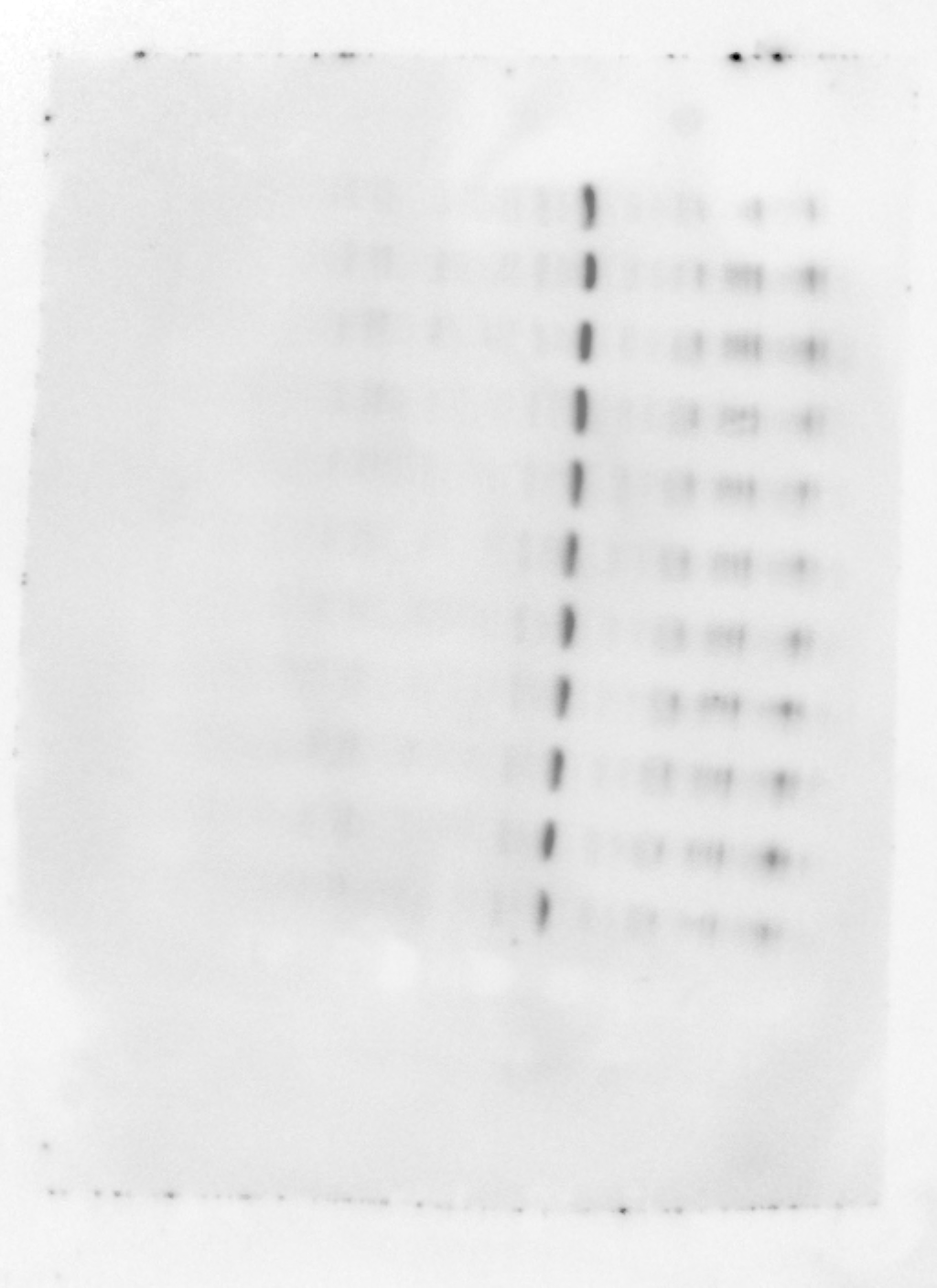

Supplement: Supplementary file 4 — Source data Fig. 2 [file 44318_2025_585_MOESM4_ESM.zip › Figure 2/2C/Western Images/ACTIN/1-13_2s_actin 2023.02.06_16.39.42_Ch.tif]

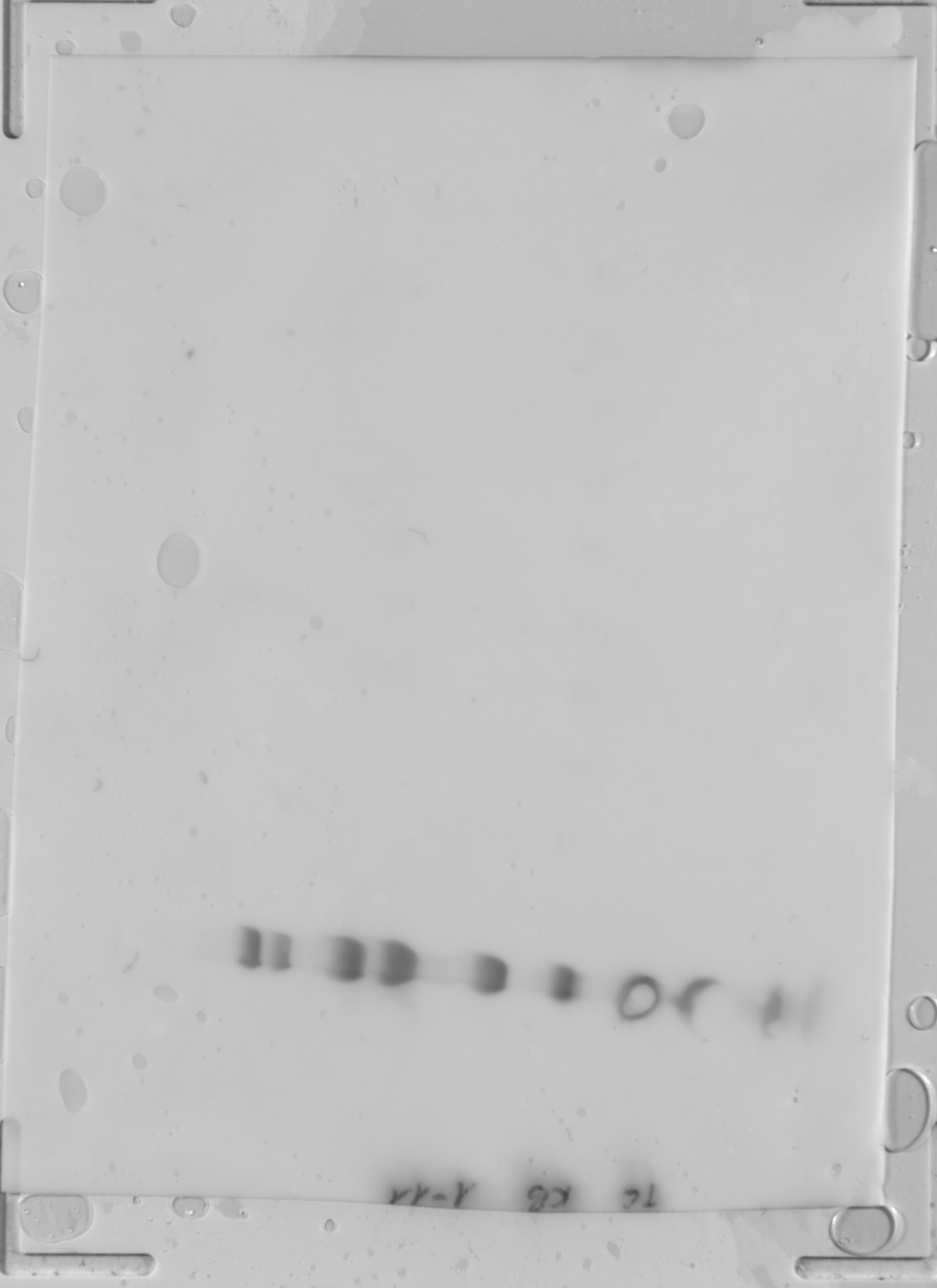

Supplement: Supplementary file 4 — Source data Fig. 2 [file 44318_2025_585_MOESM4_ESM.zip › Figure 2/2C/Western Images/ACTIN/1-13_2s_actin 2023.02.06_16.39.42_Ch-Marker.tif]

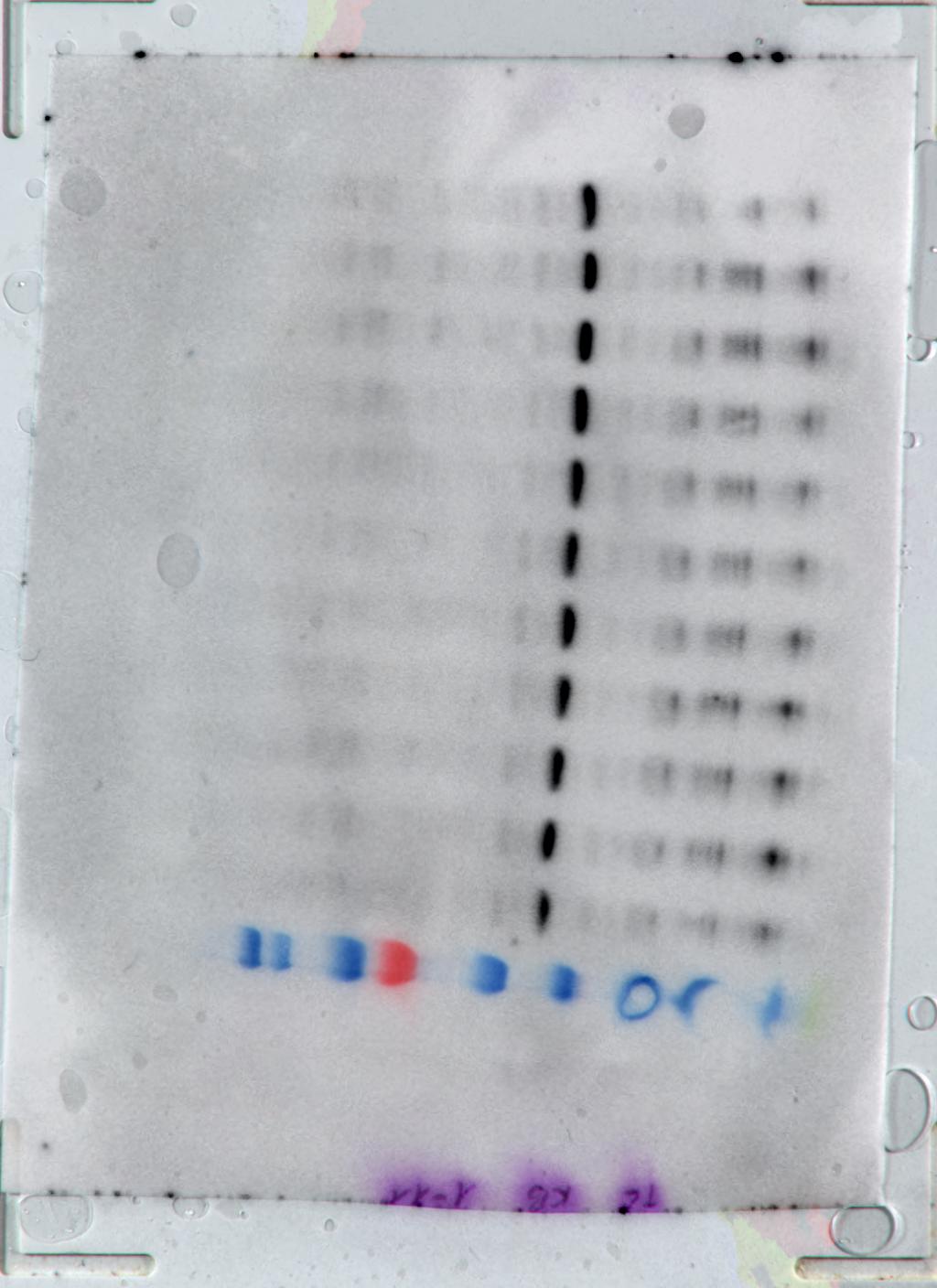

Supplement: Supplementary file 4 — Source data Fig. 2 [file 44318_2025_585_MOESM4_ESM.zip › Figure 2/2C/Western Images/ACTIN/1-13_2s_actin 2023.02.06_16.39.42_Ch+Marker.jpg]

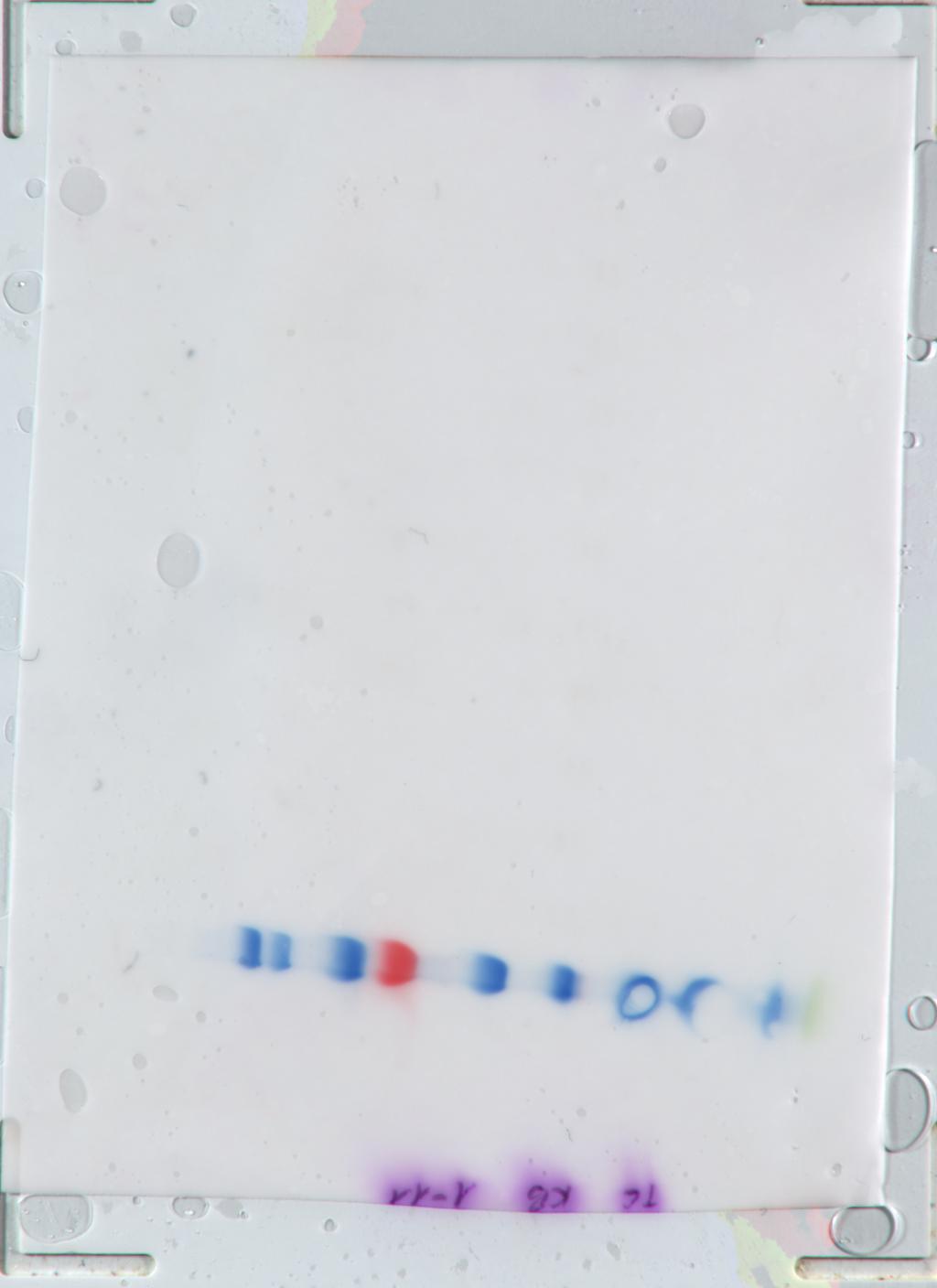

Supplement: Supplementary file 4 — Source data Fig. 2 [file 44318_2025_585_MOESM4_ESM.zip › Figure 2/2C/Western Images/ACTIN/1-13_2s_actin 2023.02.06_16.39.42_Ch-Marker.jpg]

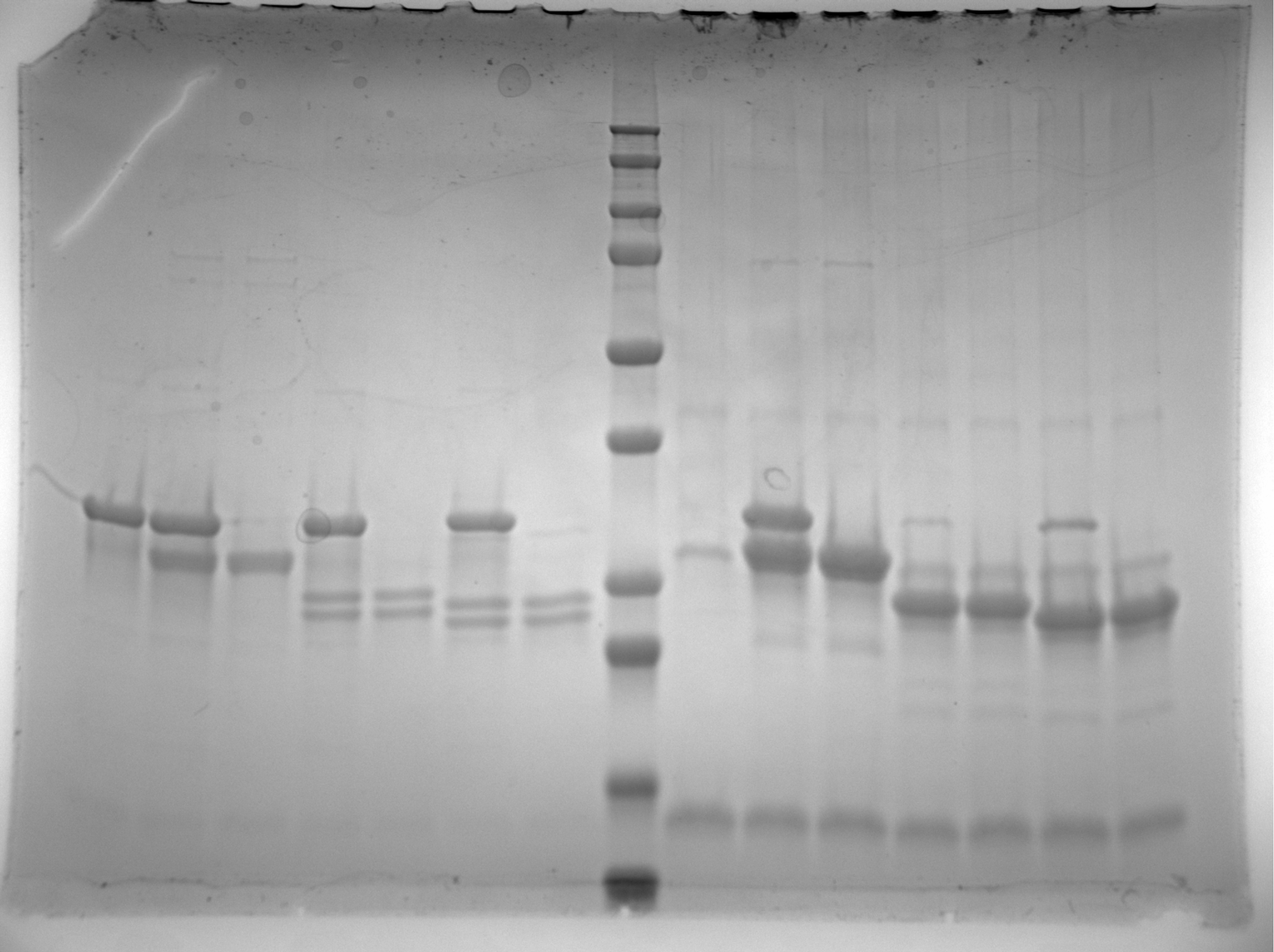

Supplement: Supplementary file 5 — Source data Fig. 3 [file 44318_2025_585_MOESM5_ESM.zip › Figure 3/3B/pulldown.tif]

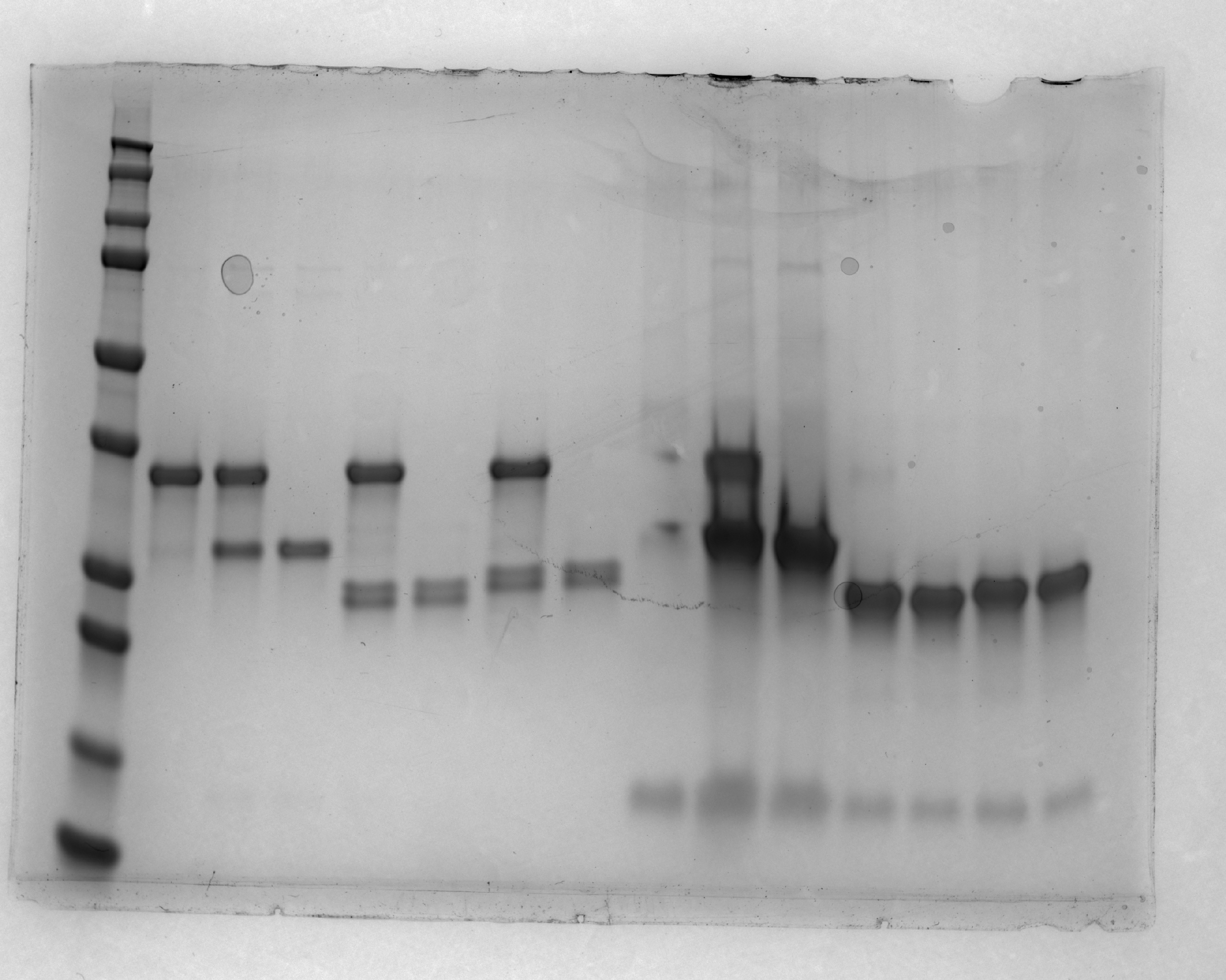

Supplement: Supplementary file 5 — Source data Fig. 3 [file 44318_2025_585_MOESM5_ESM.zip › Figure 3/3B/replicates/pulldown replicate2.tif]

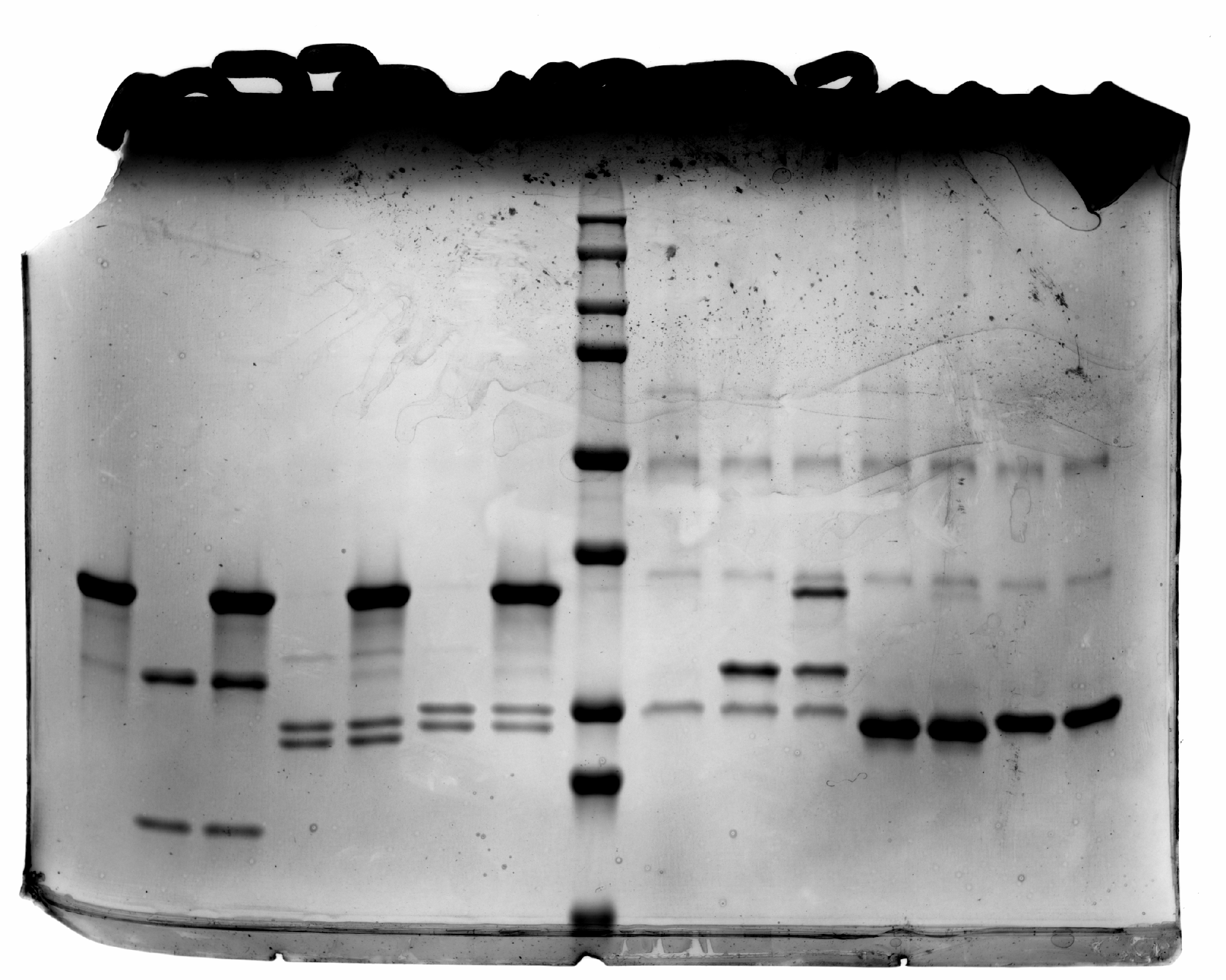

Supplement: Supplementary file 5 — Source data Fig. 3 [file 44318_2025_585_MOESM5_ESM.zip › Figure 3/3B/replicates/pulldown replicate1.tif]

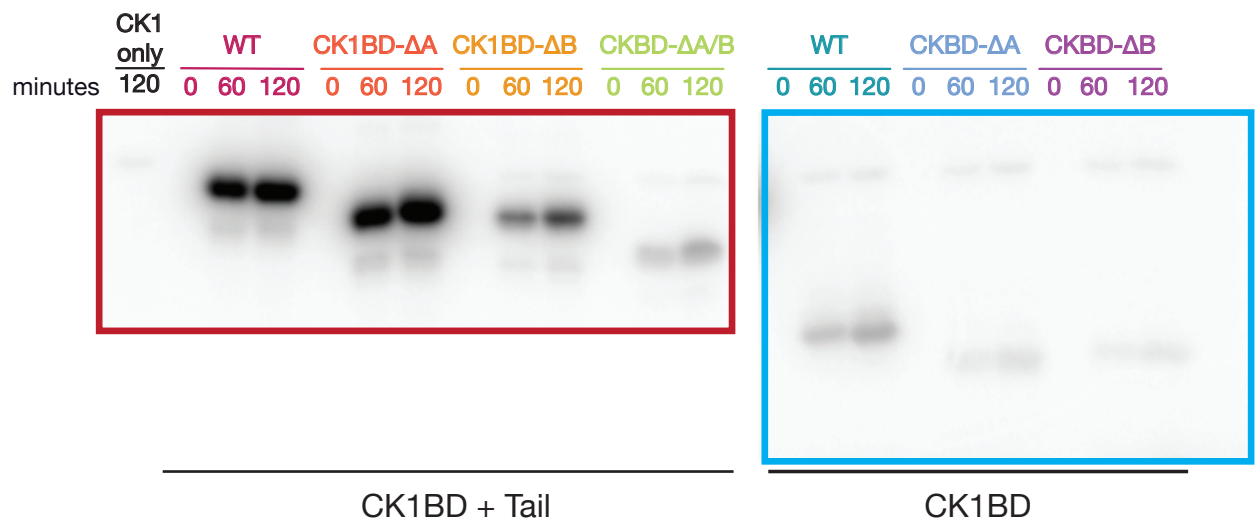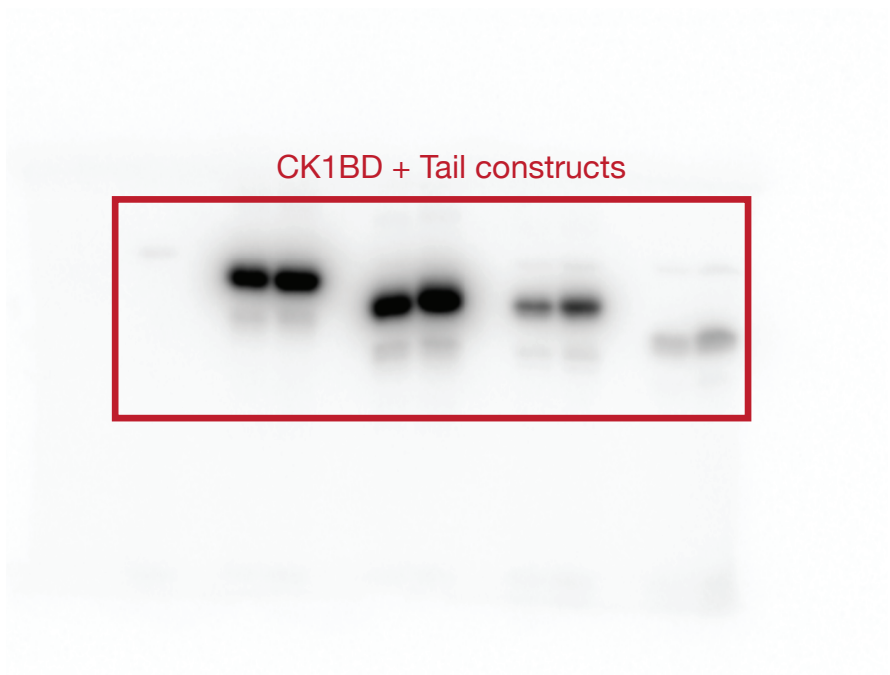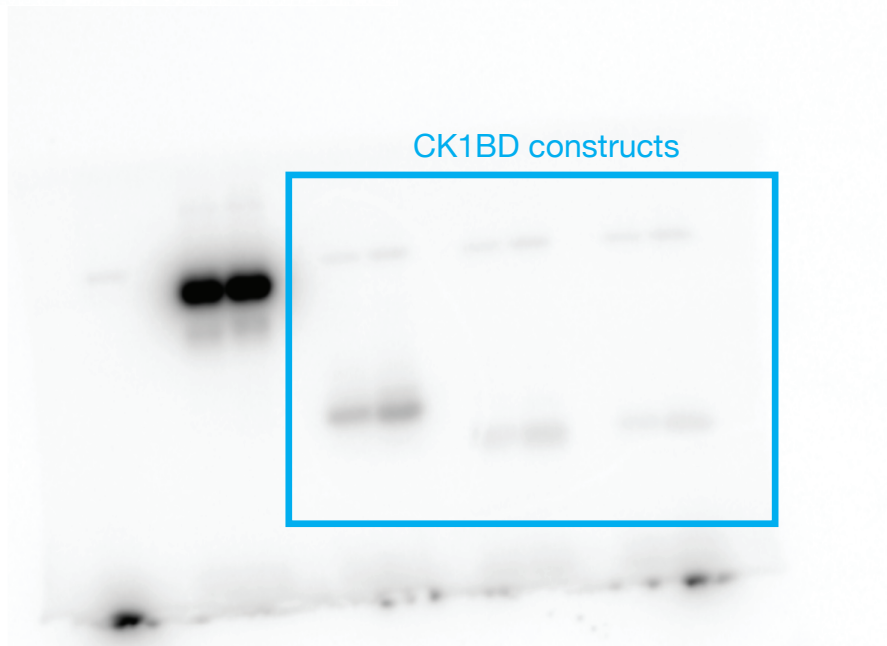

Supplement: Supplementary file 6 — Source data Fig. 4 [file 44318_2025_585_MOESM6_ESM.zip › Figure 4/4A/32P image.pdf]

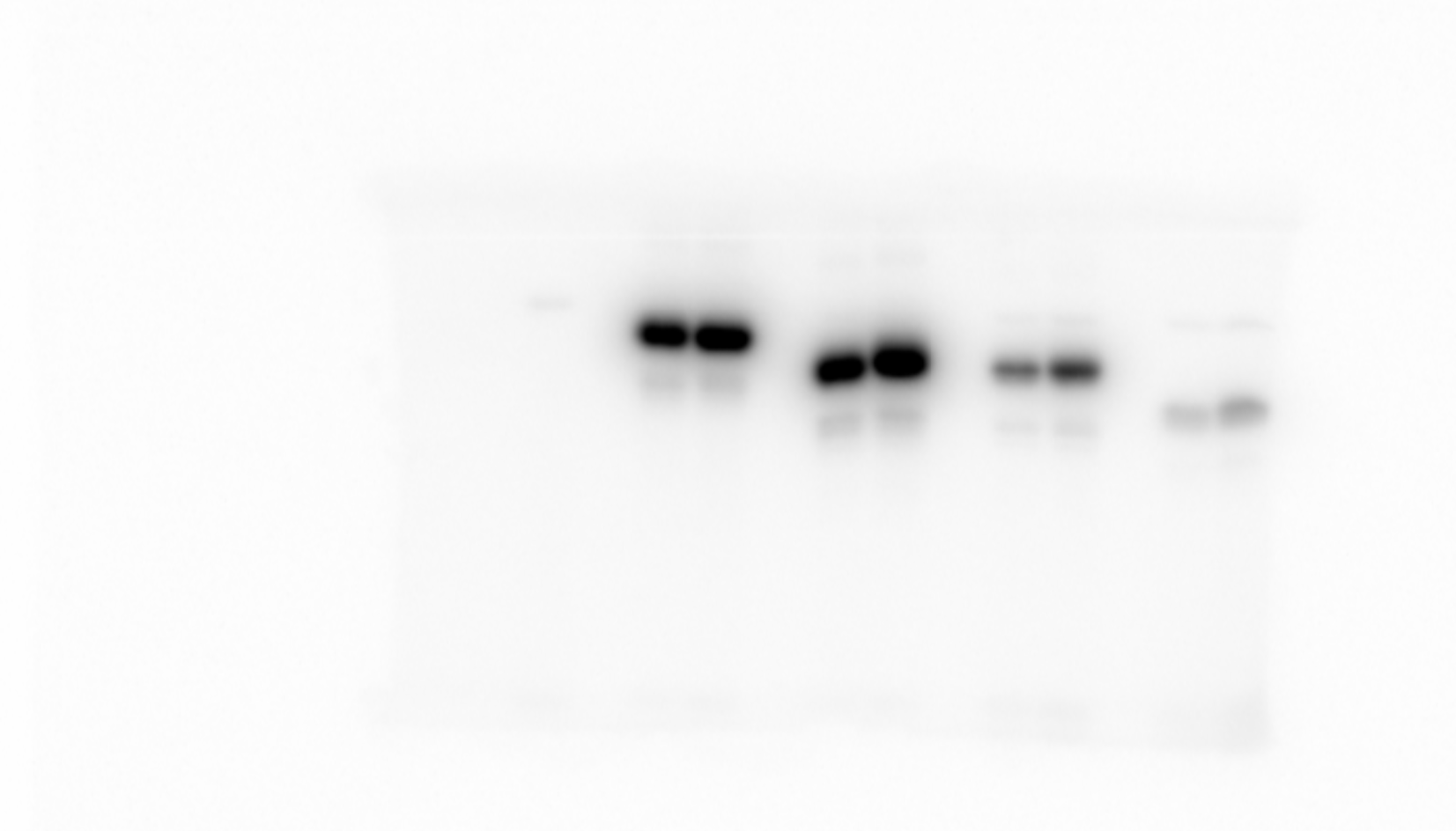

Supplement: Supplementary file 6 — Source data Fig. 4 [file 44318_2025_585_MOESM6_ESM.zip › Figure 4/4A/32P image CK1BD + Tail constructs.tif]

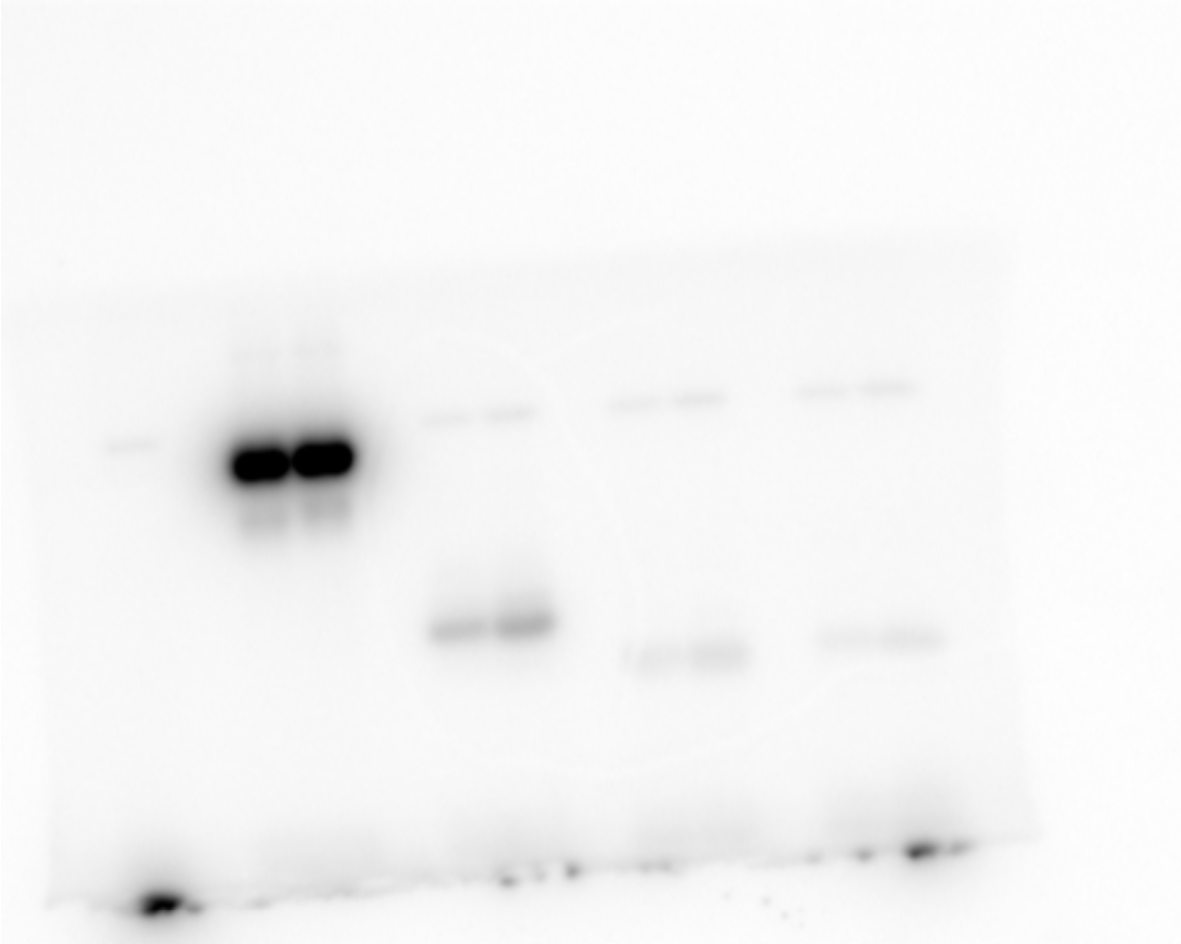

Supplement: Supplementary file 6 — Source data Fig. 4 [file 44318_2025_585_MOESM6_ESM.zip › Figure 4/4A/32P image CK1BD constructs.tif]

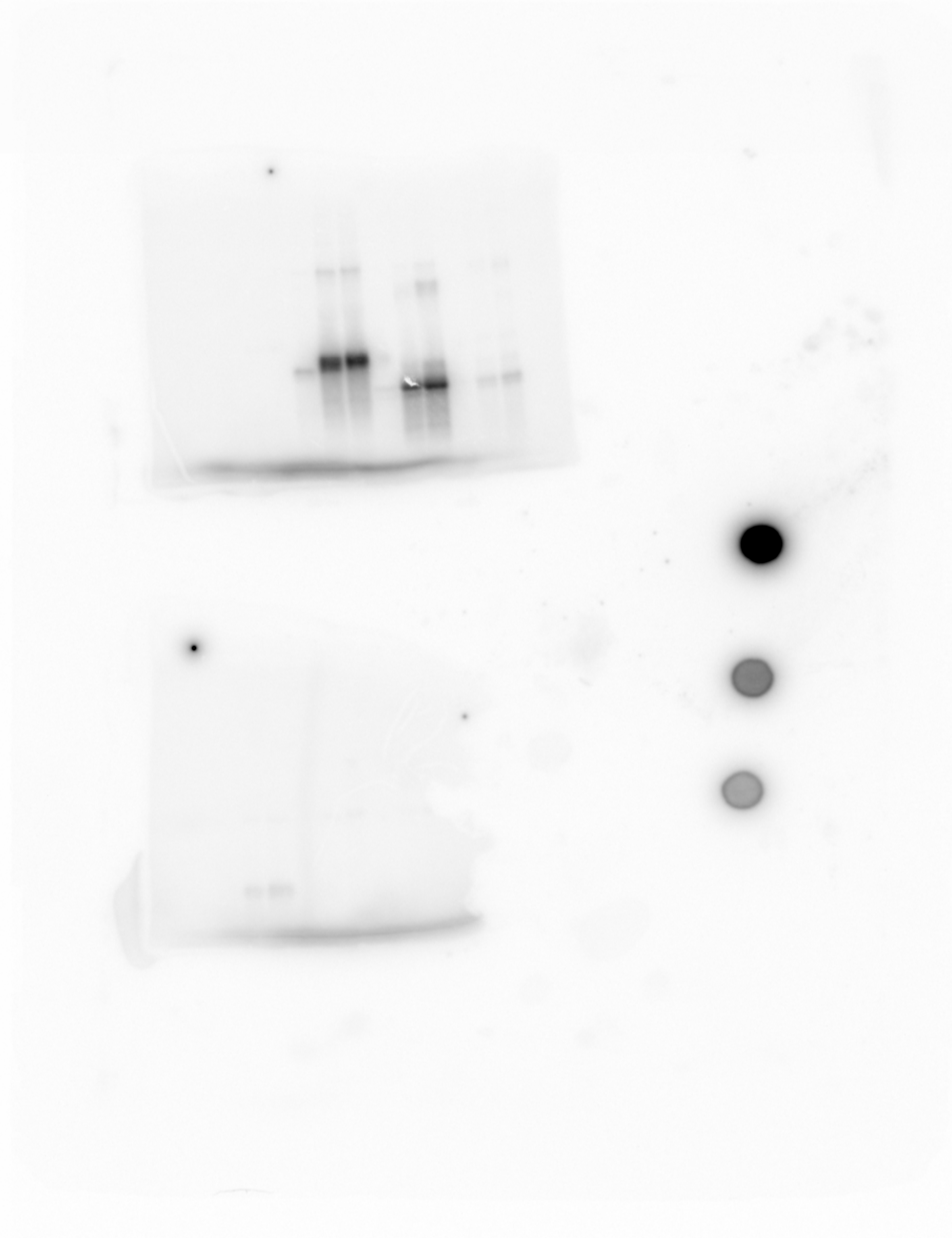

Supplement: Supplementary file 6 — Source data Fig. 4 [file 44318_2025_585_MOESM6_ESM.zip › Figure 4/4A/replicates/replicate 2/32P image replicate 2 CK1BD + Tail.tif]

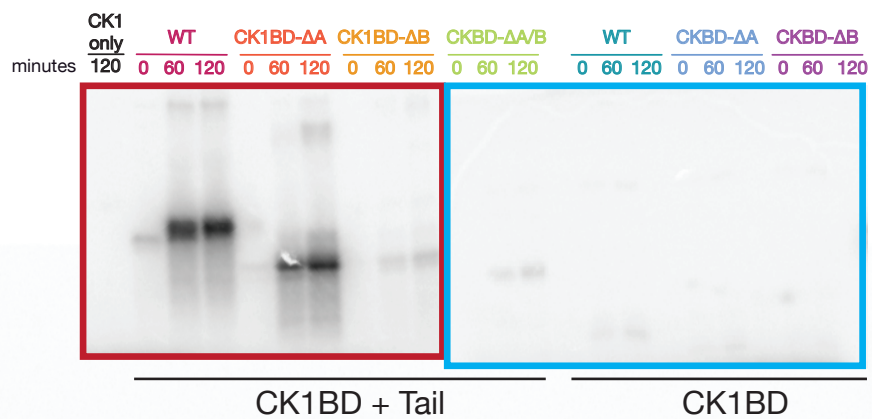

CK1BD WT, ΔA and ΔB + Tail constructs

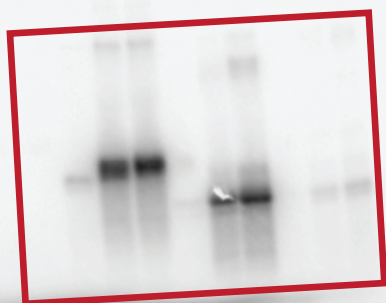

CK1BDΔA/B + Tail & CK1BD constructs

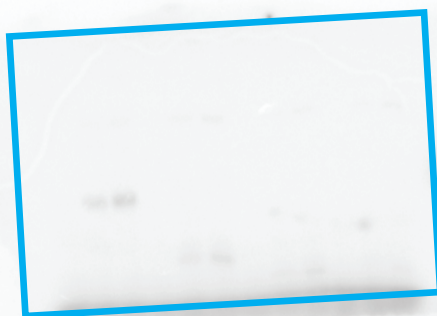

Supplement: Supplementary file 6 — Source data Fig. 4 [file 44318_2025_585_MOESM6_ESM.zip › Figure 4/4A/replicates/replicate 2/32P image replicate2.pdf]

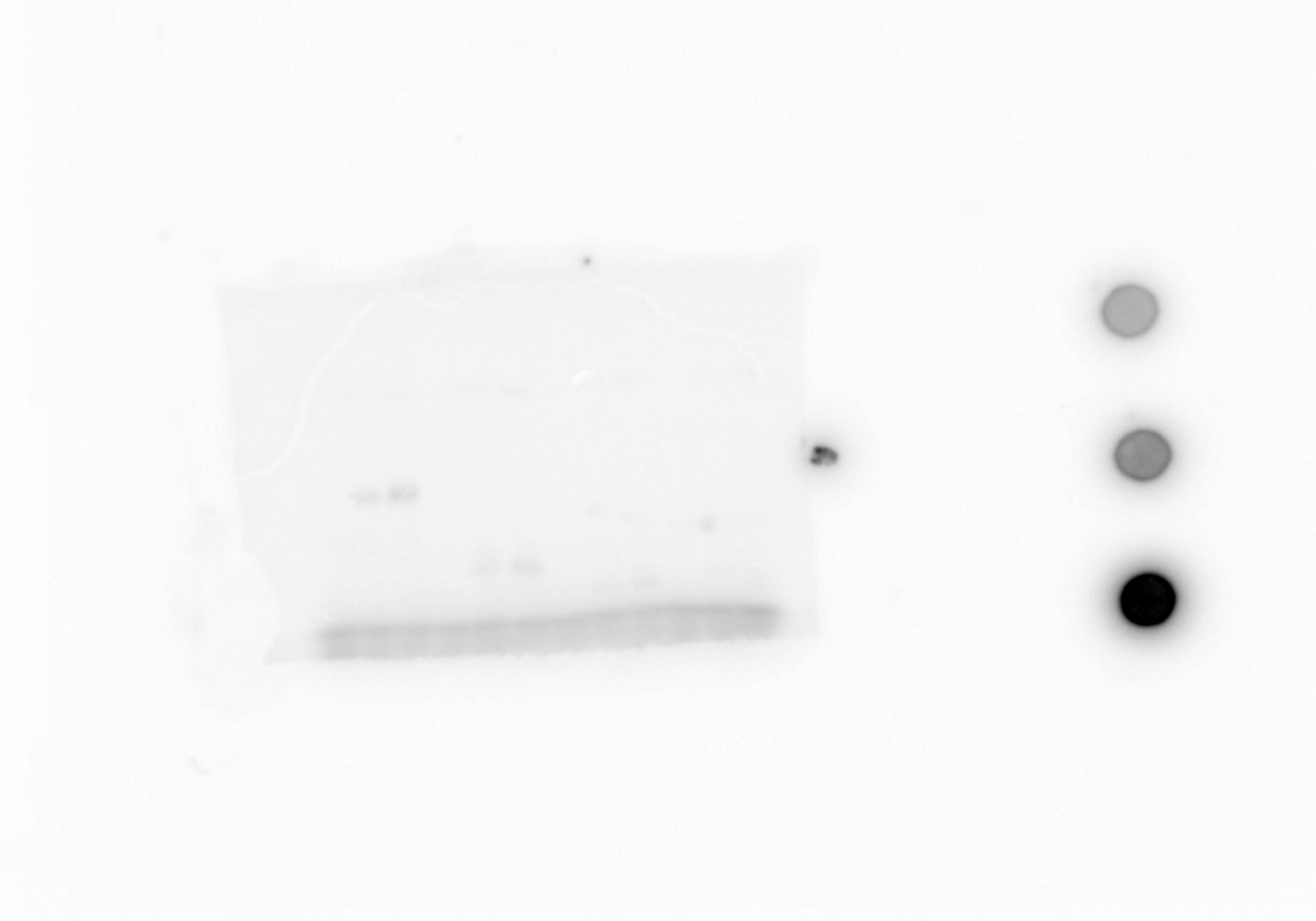

Supplement: Supplementary file 6 — Source data Fig. 4 [file 44318_2025_585_MOESM6_ESM.zip › Figure 4/4A/replicates/replicate 2/32P image replicate 2 CK1BD.tif]

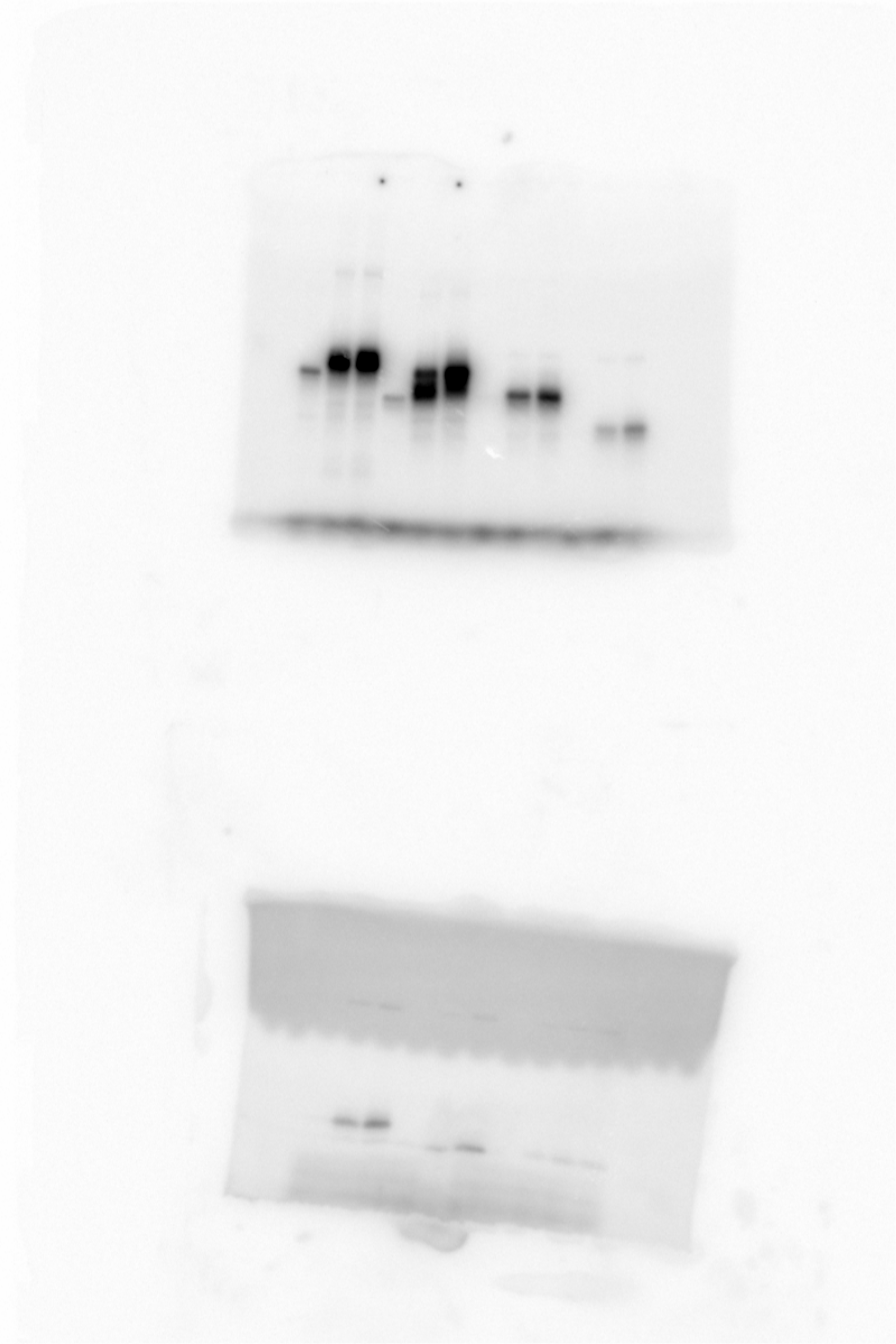

Supplement: Supplementary file 6 — Source data Fig. 4 [file 44318_2025_585_MOESM6_ESM.zip › Figure 4/4A/replicates/replicate 1/32P image replicate1.tif]

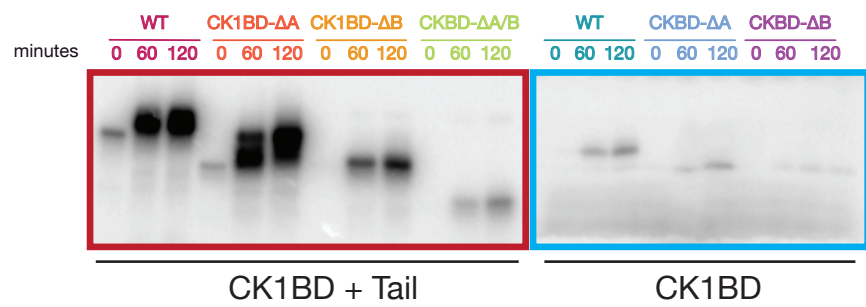

CK1BD + Tail constructs

CK1BD constructs

Supplement: Supplementary file 6 — Source data Fig. 4 [file 44318_2025_585_MOESM6_ESM.zip › Figure 4/4A/replicates/replicate 1/32P image replicate1.pdf]
