# Supplementary material for: A conserved chronobiological complex times C. elegans development
Source: EMBO J. 2025 Oct 20;44(22):6368–96. doi: 10.1038/s44318-025-00585-z (PMC12624140; doi:10.1038/s44318-025-00585-z)
Supplement: Supplementary file 5 — Source data Fig. 3 [file 44318_2025_585_MOESM5_ESM.zip › Figure 3/3B/replicates/pulldown replicates.pdf]

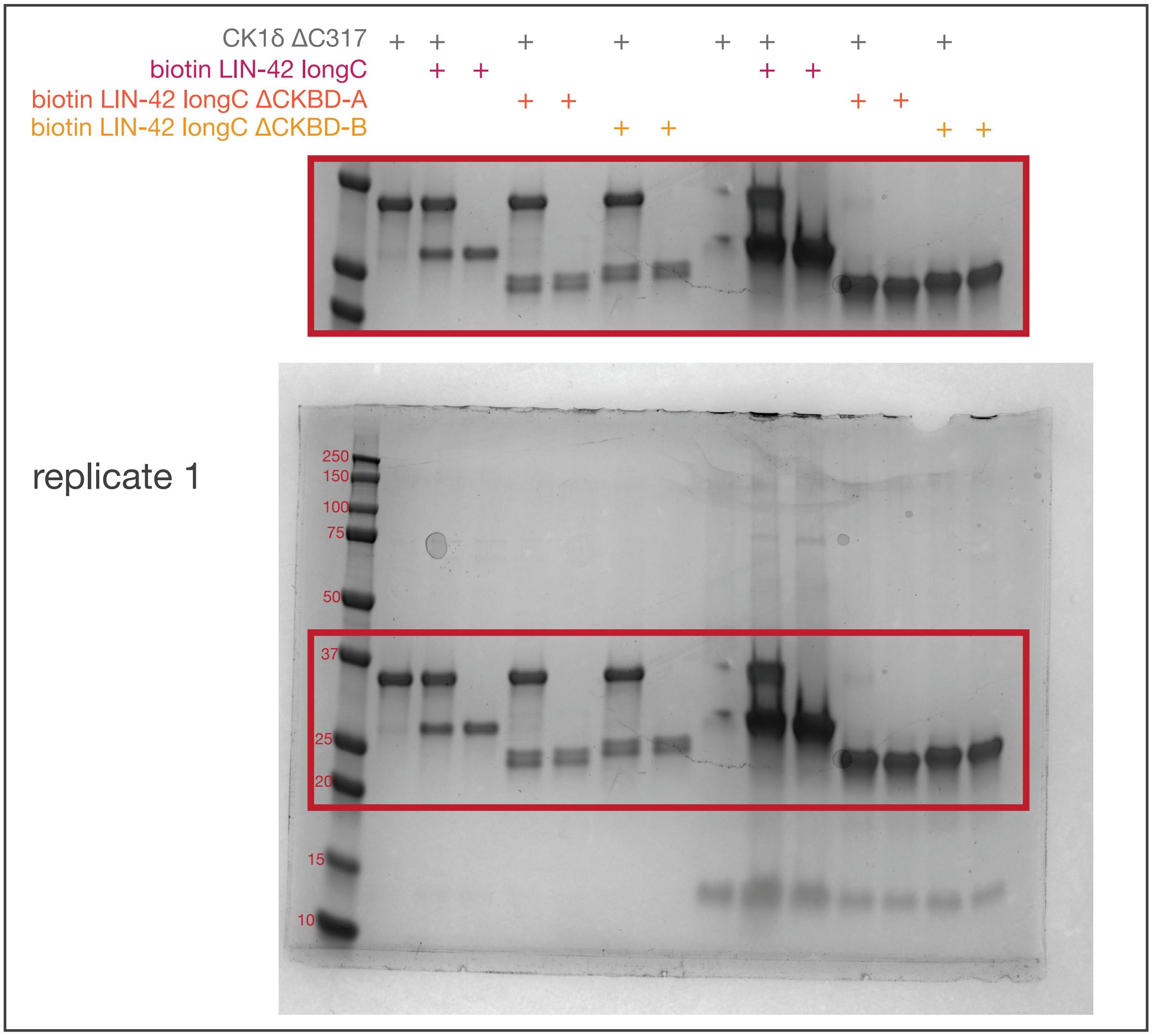
CK1δ ΔC317
biotin LIN-42 longC
biotin LIN-42 longC ΔCKBD-A
biotin LIN-42 longC ΔCKBD-B
replicate 1
250
150
100
75
50
37
25
20
15
10

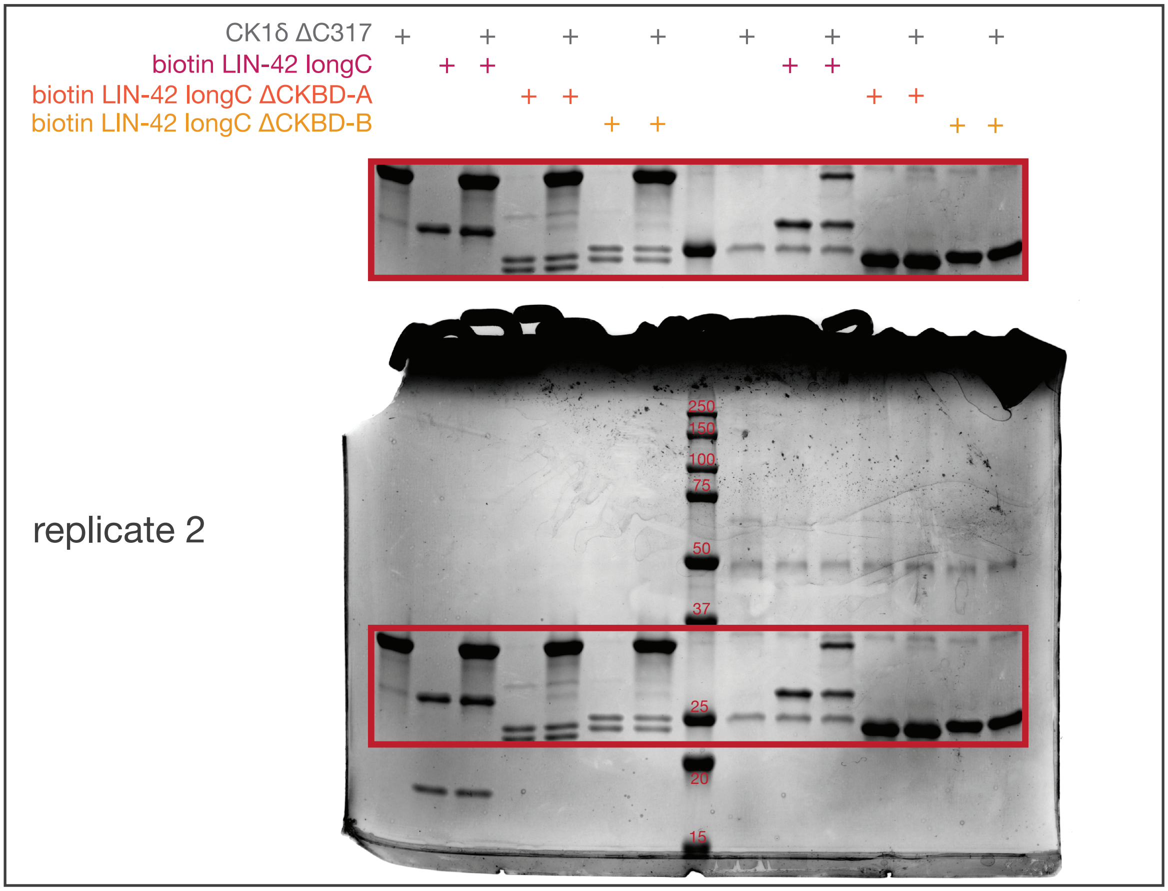
CK1δ ΔC317
biotin LIN-42 longC
biotin LIN-42 longC ΔCKBD-A
biotin LIN-42 longC ΔCKBD-B
replicate 2
250
150
100
75
50
37
25
20
15
