# Supplementary material for: A conserved chronobiological complex times C. elegans development
Source: EMBO J. 2025 Oct 20;44(22):6368–96. doi: 10.1038/s44318-025-00585-z (PMC12624140; doi:10.1038/s44318-025-00585-z)
Supplement: Supplementary file 12 — Expanded View Figures [file 44318_2025_585_MOESM12_ESM.pdf]

Expanded View Figures

Figure EV1. Larval stage durations for *lin-42* mutant animals.

Boxplots showing durations (in hours) for (**A**) larval stages (**B**) molts and (**C**) intermolts from luciferase assay. Wild type in white, *lin-42(n1089)* in dark grey, *lin-42(ok2385)* in light grey, *lin-42(wrd67[ΔPAS])* in blue and *lin-42(wrd63[ΔCK1BD])* in green. Statistics were done using the Mann–Whitney *U* test. Stars indicate the significance of difference between the *Wt* strain and the different *lin-42* mutant animals: *$P < 0.05$, **$P < 0.01$, ***$P < 0.001$, ****$P < 0.0001$. (**D**) Bar plot showing the number of molts from the luciferase assay of the indicated genotypes. Boxplots were generated using the boxplot function in python's seaborn package (v0.13.2) using the default options (center = median, boxes represent values within the 0.25 (Q1) and 0.75 (Q3) quantiles (the interquantile distance or IQR), whiskers represent values within Q1 - 1.5IQR and Q3 + 1.5IQR, and extrema are the minima and maxima for each condition). Two biological replicates were performed for each strain.

© The Author(s)

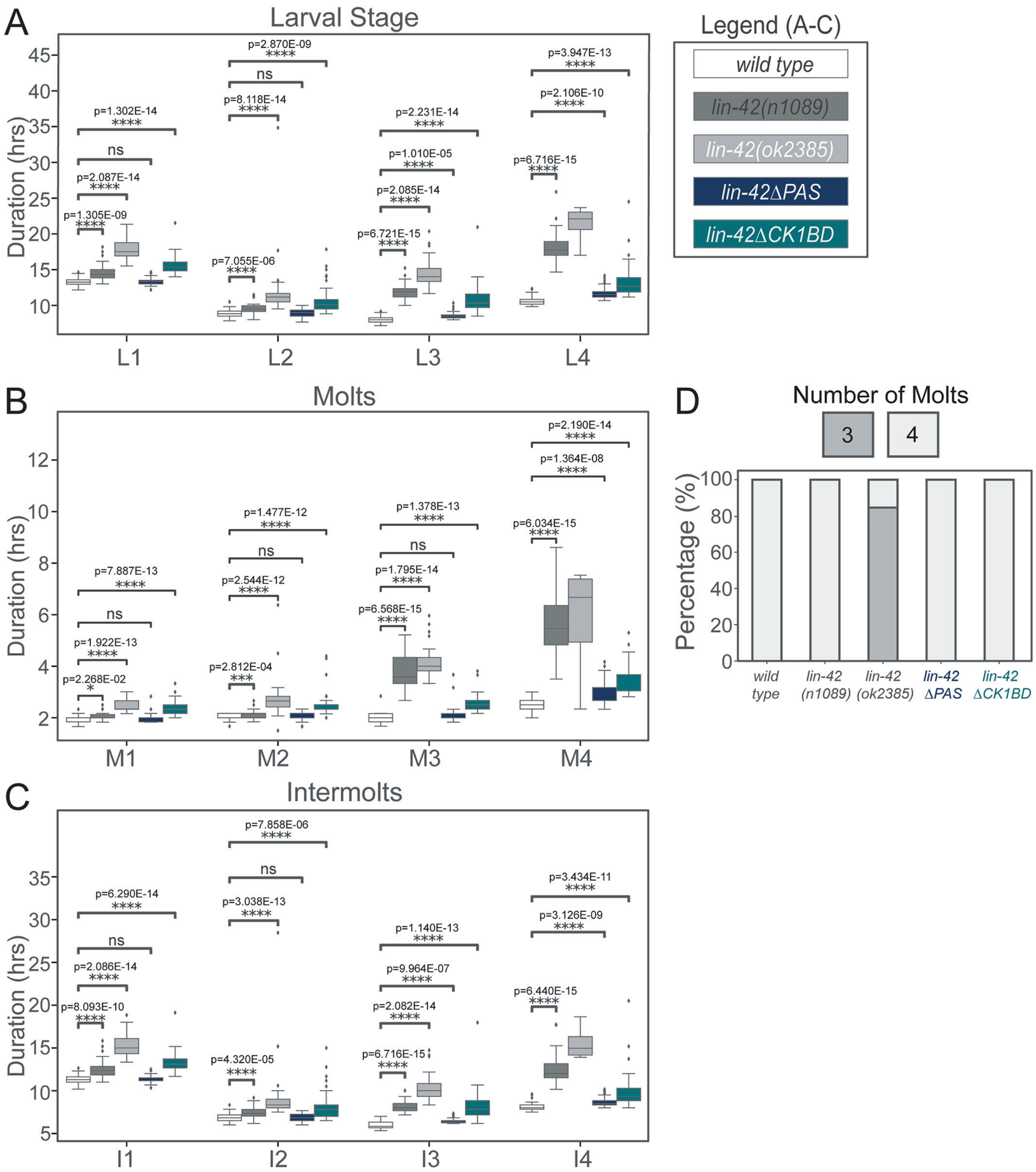
A
Larval Stage
Duration (hrs)
L1
L2
L3
L4
Legend (A-C)
wild type
lin-42(n1089)
lin-42(ok2385)
lin-42ΔPAS
lin-42ΔCK1BD
B
Molts
M1
M2
M3
M4
C
Intermolts
I1
I2
I3
I4
D
Number of Molts
3
4
Percentage (%)
wild type
lin-42 (n1089)
lin-42 (ok2385)
lin-42 ΔPAS
lin-42 ΔCK1BD

© The Author(s)

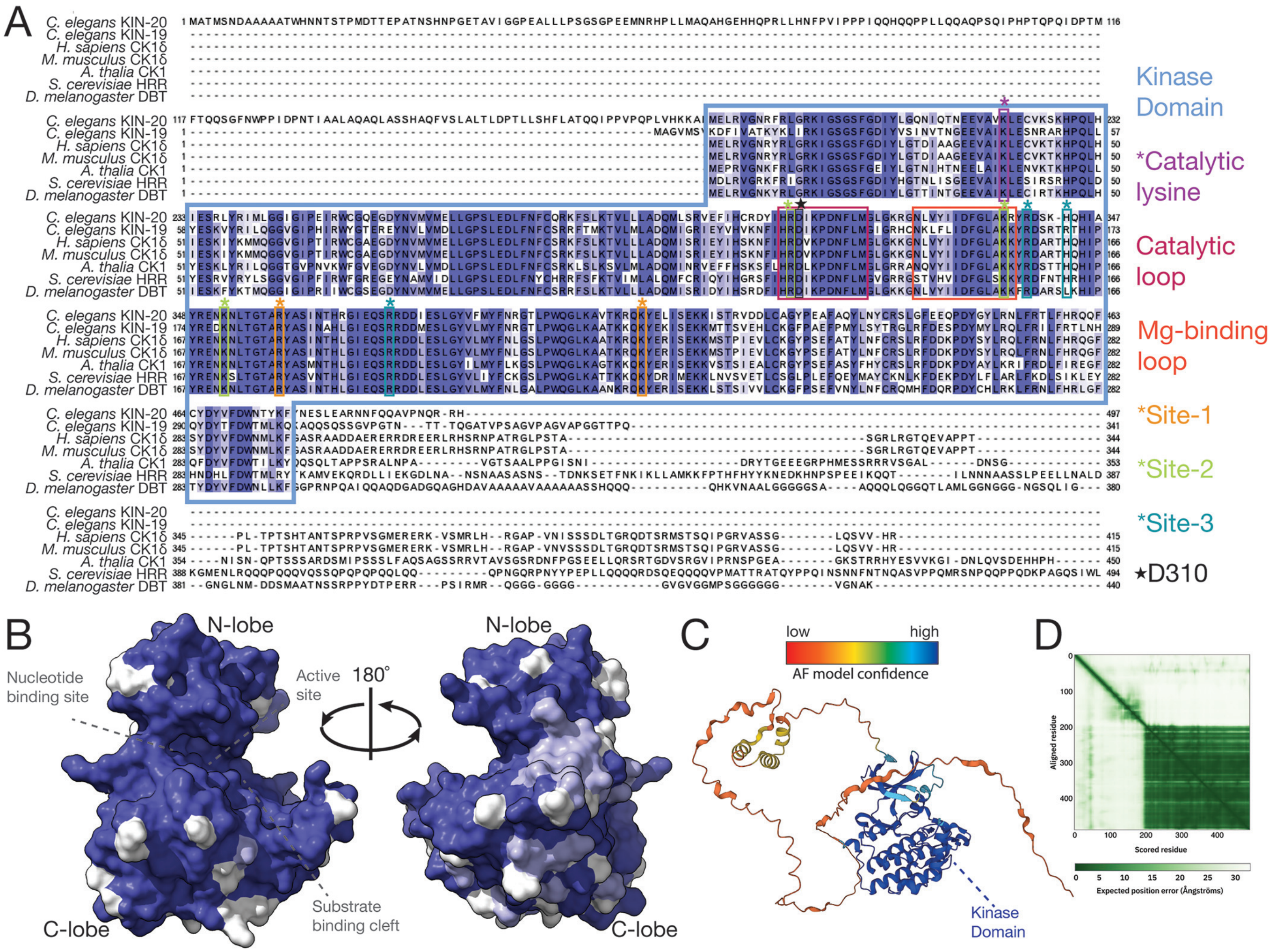

Figure EV2. Conservation and structural prediction of *C. elegans* KIN-20.

(**A**) Sequence alignment of *C. elegans* KIN-20 and KIN-19, *H. sapiens* CK1δ, *M. musculus* CK1δ, *A. thalia* CK1, *S. cerevisiae* HRR25 (HRR), and *D. melanogaster* Doubletime (DBT). Important enzymatic sequence features are boxed; full kinase domain (blue box - 79% identical between KIN-20 and human CK1δ), catalytic lysine (purple - conserved in KIN-20), catalytic loop (pink - 100% conserved between KIN-20 and human CK1δ), Magnesium (Mg)-binding loop (orange - 12 out of 13 residues conserved between KIN-20 and human CK1), anion coordination sites 1 (yellow), 2 (green), and 3 (blue) (all conserved in KIN-20). (**B**) Crystal structure of *H. sapiens* CK1δ (PDB 6pxo). Dark blue indicates residue is conserved in KIN-20; residues that diverge are highlighted in pale blue (similar amino acid) or white (not conserved). (**C**) AlphaFold structural prediction of KIN-20 (https://alphafold.ebi.ac.uk/entry/A8X4B3) colored by the model confidence. (**D**) KIN-20 AlphaFold plot of predicted aligned error.

© The Author(s)

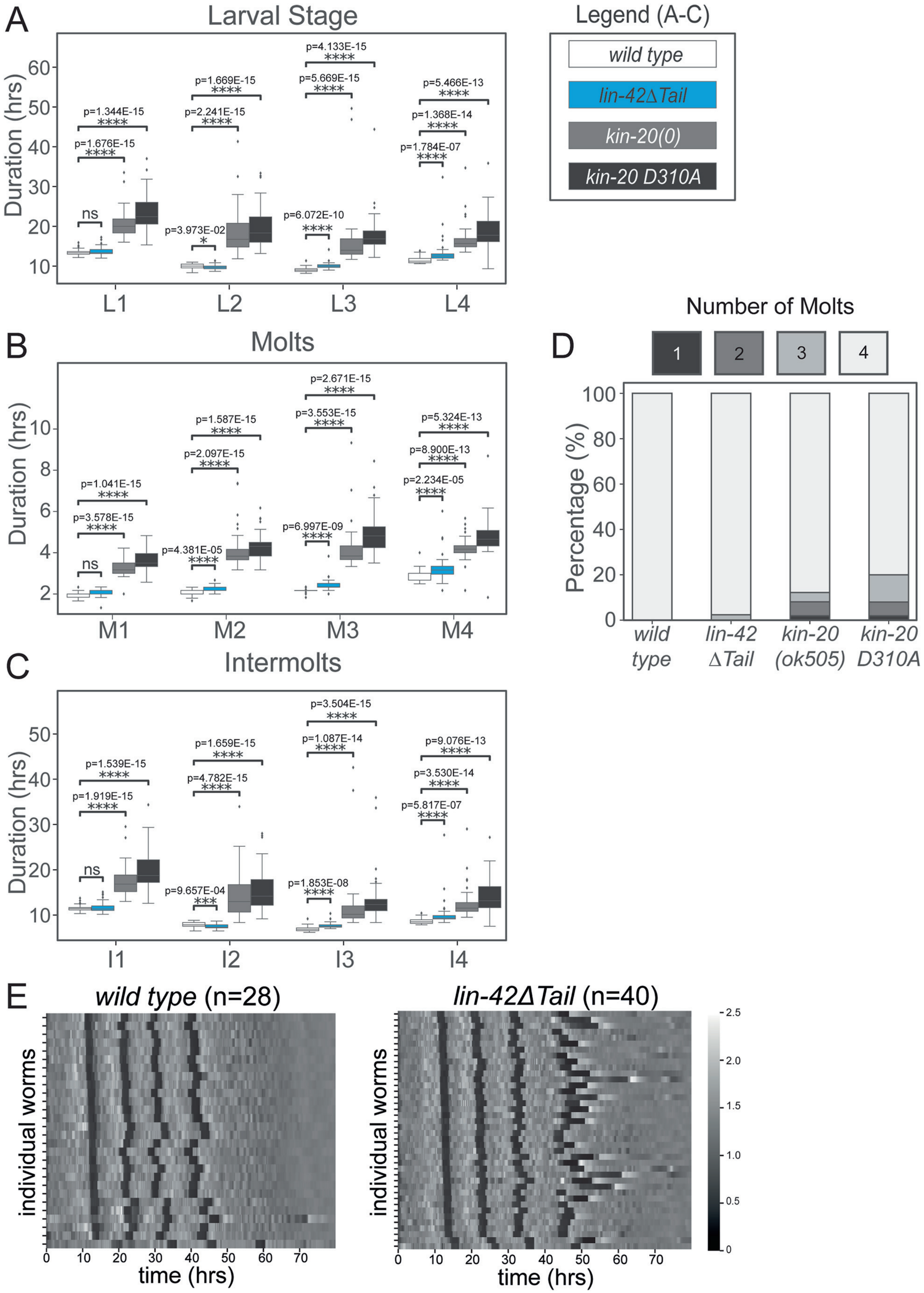
A
Larval Stage
Duration (hrs)
60
50
40
30
20
10
p=1.344E-15
p=1.676E-15
ns
p=1.669E-15
p=2.241E-15
p=3.973E-02
p=4.133E-15
p=5.669E-15
p=6.072E-10
p=5.466E-13
p=1.368E-14
p=1.784E-07
L1
L2
L3
L4
Legend (A-C)
wild type
lin-42ΔTail
kin-20(0)
kin-20 D310A
B
Molts
Duration (hrs)
10
8
6
4
2
p=1.041E-15
p=3.578E-15
ns
p=1.587E-15
p=2.097E-15
p=4.381E-05
p=2.671E-15
p=3.553E-15
p=6.997E-09
p=5.324E-13
p=8.900E-13
p=2.234E-05
M1
M2
M3
M4
Number of Molts
D
1
2
3
4
Percentage (%)
100
80
60
40
20
0
wild type
lin-42 ΔTail
kin-20 (ok505)
kin-20 D310A
C
Intermolts
Duration (hrs)
50
40
30
20
10
p=1.539E-15
p=1.919E-15
ns
p=1.659E-15
p=4.782E-15
p=9.657E-04
p=3.504E-15
p=1.087E-14
p=1.853E-08
p=9.076E-13
p=3.530E-14
p=5.817E-07
I1
I2
I3
I4
E
wild type (n=28)
individual worms
0 10 20 30 40 50 60 70
time (hrs)
lin-42ΔTail (n=40)
individual worms
0 10 20 30 40 50 60 70
time (hrs)
2.5
2.0
1.5
1.0
0.5
0

Figure EV3. Larval stage durations for *kin-20 and lin-42* mutant animals.

Boxplots showing durations (in hours) for (**A**) larval stages, (**B**) molts and (**C**) intermolts from luciferase assay. Wild type (*Wt*) in white, *lin-42(wrd107[ΔTail])* in blue, *kin-20(0)* in light grey, *kin-20(xe355[D310A])* in dark grey. Statistics were done using the Mann–Whitney *U* test. Stars indicate the significance of difference between the *Wt* strain and the different *lin-42* and *kin-20* mutant animals: *$P < 0.05$, **$P < 0.01$, ***$P < 0.001$, ****$P < 0.0001$. (**D**) Bar plot showing the number of molts detected in the assay in percentage of animals. (**E**) Luciferase replicate of *lin-42(wrd107[ΔTail]*. Heatmaps showing trend-corrected luminescence traces from the indicated genotype. Each horizontal line represents one animal. Traces are sorted to the entry of the first molt. Darker color indicates low luminescence signal and corresponds to the molts. Boxplots were generated using the boxplot function in Python's seaborn package (v0.13.2) using the default options (center = median, boxes represent values within the 0.25 (Q1) and 0.75 (Q3) quantiles (the interquantile distance or IQR), whiskers represent values within Q1 - 1.5IQR and Q3 + 1.5IQR, and extrema are the minima and maxima for each condition). Two biological replicates were performed for each strain. Source data are available online for this figure.

 © The Author(s)

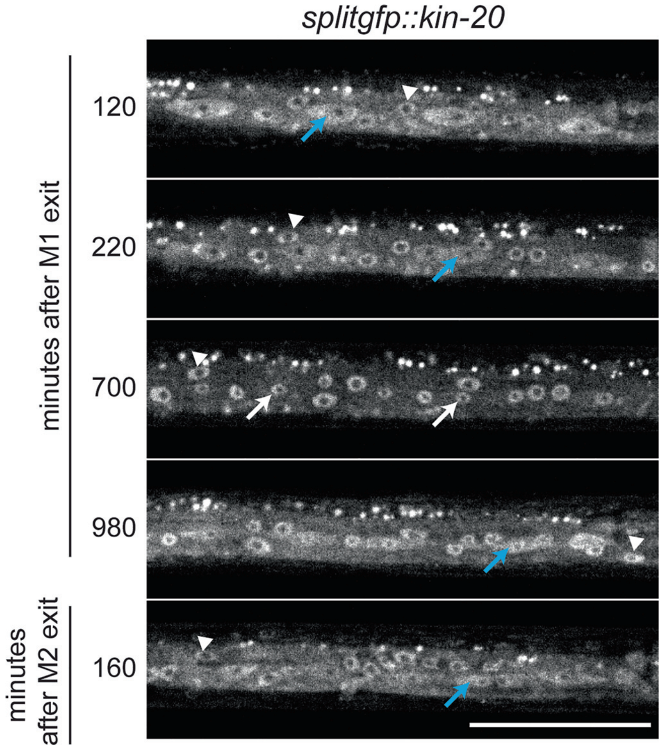

Figure EV4. KIN-20 dynamics during L2 - L3 stage.

Time-lapse microscopy images of a single *splitgfp::kin-20* larva (*bchSi84[eft-3p::gfp1-10(codon-optimized)::tbb-2 3UTR]); kin-20[(xe354)[3xflag::4xgfp11]])* followed in a microfluidics device over time. Time indicated in minutes after molt 1 (M1) or molt 2 (M2) exit. Arrows indicate seam cell nuclear (white) and cytoplasmic (blue) localization; white arrowheads indicate nuclear hyp7 localization. Scale bar = 50 µm. This experiment was performed once. Source data are available online for this figure.

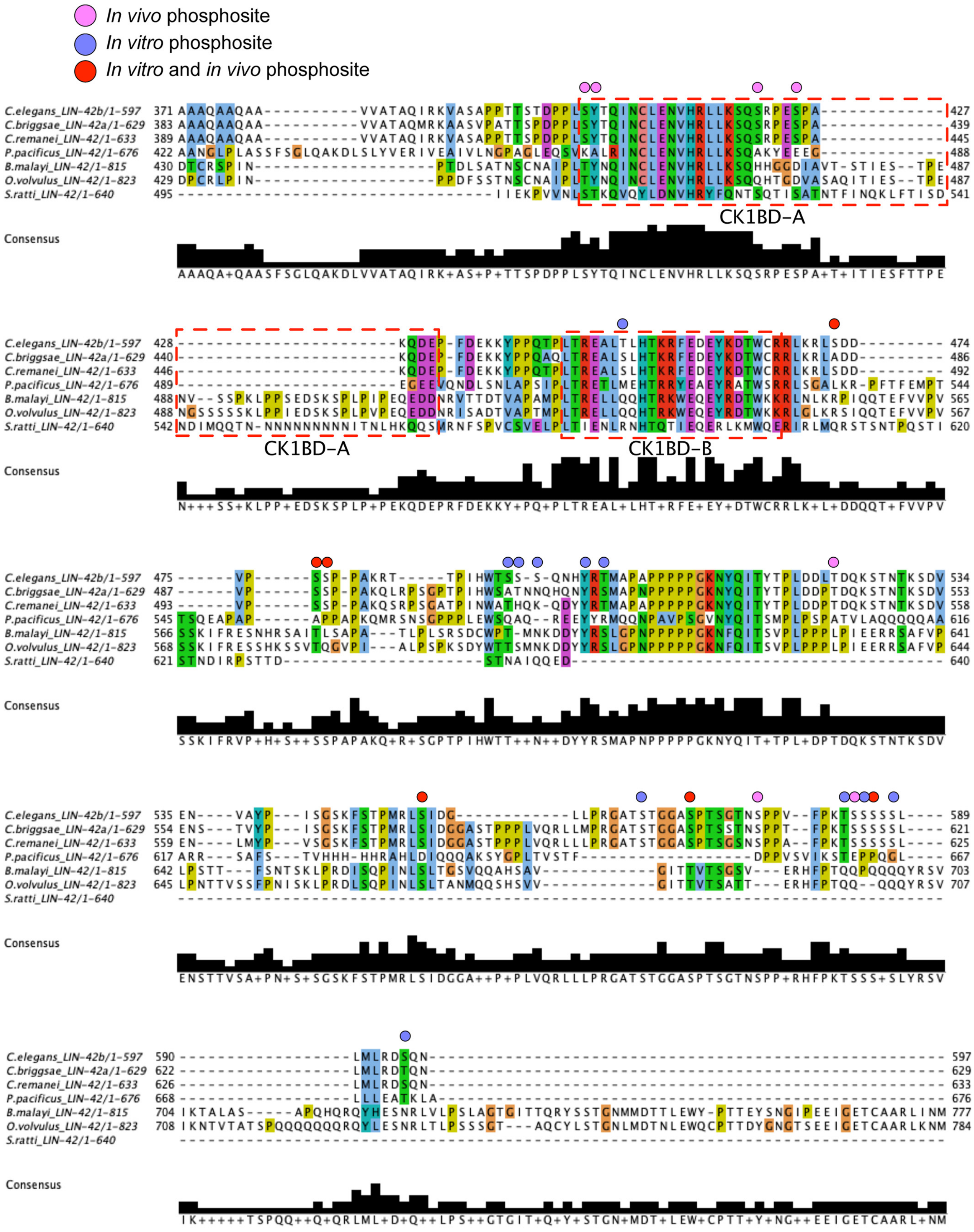
In vivo phosphosite
In vitro phosphosite
In vitro and in vivo phosphosite
C.elegans_LIN-42b/1-597
C.briggsae_LIN-42a/1-629
C.remanei_LIN-42/1-633
P.pacificus_LIN-42/1-676
B.malayi_LIN-42/1-815
O.volvulus_LIN-42/1-823
S.ratti_LIN-42/1-640
CK1BD-A
CK1BD-B
Consensus

© The Author(s)

Figure EV5. Alignment of nematode LIN-42 protein sequences.

LIN-42 homologs from the indicated nematode species were aligned using Clustal Omega. The length in amino acids of each homolog follows the species and homolog name. To the left and right of the alignment are amino acid positions of the end residues for each protein. Blue shading indicates conserved sequences and the histogram at the bottom depicts the degree of conservation with a consensus sequence listed below. The positions of the *C. elegans* CK1BD-A and CK1BD-B motifs are indicated. The location of the phosphosites found in our in vivo, in vitro and both datasets are indicated.

© The Author(s)
